# Experimental evidence of electroacupuncture in ALS mouse models: a systematic review and meta-analysis

**DOI:** 10.3389/fneur.2026.1780176

**Published:** 2026-07-01

**Authors:** Mengwan Hu, Lei You, Xuezhuo Zhang, Zhibo Xuan, Sijia Ma, Xian Wu

**Affiliations:** 1 Graduate School, Heilongjiang University of Chinese Medicine, Harbin, China; 2 Department of Acupuncture, Heilongjiang Academy of Chinese Medicine Sciences, Harbin, China

**Keywords:** amyotrophic lateral sclerosis, animal experiments, electroacupuncture, meta-analysis, motor neuron

## Abstract

**Objective:**

This study aimed to systematically evaluate the therapeutic efficacy of electroacupuncture (EA) in amyotrophic lateral sclerosis (ALS) and to elucidate the underlying neurobiological mechanisms by synthesizing preclinical evidence.

**Methods:**

According to the PICOS principle, relevant studies were searched in the following databases: PubMed, Web of Science, Embase, Cochrane Library, Scopus, and CNKI. Search terms and strategies were determined based on MeSH terms. The methodological quality of the included studies was assessed using the SYRCLE’s Risk of Bias tool and the CAMARADES checklist. Meta-analysis was performed using Stata 15.0 and Rstudio software.

**Results:**

Seventeen studies involving 372 animals were included. The quality scores of the included studies ranged from 5 to 8, with an average score of 7. The meta-analysis of the primary outcome, the rotarod test score, showed a significant improvement in the EA group compared to the control group [SMD = 3.31, 95% CI (2.05, 4.57), *Z* = 5.151, *p* < 0.001], indicating that EA can enhance motor function in ALS mice. Regarding secondary outcomes, EA intervention alleviated neuroinflammation, promoted neuronal survival, improved axonal regeneration inhibition, and stabilized RNA metabolism homeostasis. Consequently, it slowed disease progression, improved motor performance, prolonged survival time, and effectively protected motor neurons at the histopathological level (*p* < 0.05). These findings underscore the potential of EA as a promising multimodal therapeutic strategy for ALS. For the heterogeneity observed in the rotarod test, sensitivity analysis, subgroup analysis, and meta-regression did not identify its source. However, potential publication bias was detected, which might contribute to the heterogeneity. The heterogeneity for other outcome measures might originate from differences in stimulation parameters (e.g., waveform), acupoint selection, or treatment duration.

**Conclusion:**

This meta-analysis demonstrates that EA confers significant neuroprotective benefits in preclinical ALS models, primarily through multi-target modulation of key pathological processes such as neuroinflammation, aberrant cell death signaling, and RNA metabolism. These preclinical findings underscore the potential of electroacupuncture as a complementary neuroprotective strategy and warrant further investigation in rigorous clinical trials.

**Systematic review registration:**

https://www.crd.york.ac.uk/PROSPERO/view/CRD420251229183.

## 1 Introduction

ALS is a progressive and fatal neurodegenerative disease characterized by the selective loss of motor neurons in the brain and spinal cord, leading to muscle weakness, atrophy, and ultimately death from respiratory failure in patients (1). The disease imposes a severe dual burden on both society and families, encompassing health and economic dimensions. On one hand, patients suffer from a short survival period and a markedly poor quality of life (2). On the other hand, direct medical costs escalate exponentially with disease progression, particularly in the advanced stages requiring respiratory support and comprehensive care, significantly exceeding those of the non-ALS population, which underscores the substantial burden of the disease (3). Current clinical strategies are primarily focused on limited disease-modifying and symptomatic support. While drugs like riluzole and edaravone can modestly delay disease progression, they cannot reverse its course and show no significant effect on improving quality of life (4). Although respiratory support can prolong life, it does not address the core issue of neuronal degeneration (5). Consequently, existing therapies face limitations in terms of efficacy, safety, and durability. The imbalance between the high economic burden and the low therapeutic efficacy makes the exploration of more suitable alternative or adjunctive strategies particularly urgent (3).

Acupuncture, a traditional Chinese therapeutic modality with a long history, is gaining increasing international recognition and has been applied in numerous countries (6). EA is a modern adaptation developed from this foundation, which involves applying controlled microcurrents through connected traditional filiform needles. This approach integrates traditional empirical knowledge with modern electrophysiological principles, forming the research basis for its use as a neuromodulation tool (7). Compared to manual acupuncture, a key distinction of EA lies in its ability to deliver sustained, stable, and precisely controllable stimulation parameters (intensity, frequency, duration), ultimately enabling synergistic multi-target interventions on the nervous system (8). Modern research preliminarily reveals that the potential mechanisms of action of EA show a high degree of alignment with key pathological processes in ALS. Firstly, EA can modulate the release and balance of neurotransmitters such as glutamate and Gamma-Aminobutyric Acid (GABA), potentially effectively countering the core “glutamate excitotoxicity” damage in ALS (9). Secondly, EA can promote the expression of various neurotrophic factors, including brain-derived neurotrophic factor (BDNF), enhancing synaptic plasticity and providing potential support for damaged motor neurons (10). Furthermore, research by Xie et al. (11) suggests that electroacupuncture may also exert anti-neuroinflammatory effects by modulating microglial activity. These mechanisms indicate that EA possesses the unique potential for synergistic intervention across multiple pathogenic pathways in ALS.

Therefore, based on its multi-target characteristics and existing neuroscientific evidence, a systematic evaluation of the effects of EA intervention in ALS and elucidation of its molecular and cellular mechanisms hold significant scientific value for understanding EA’s role in treating ALS. Moreover, it holds promise for opening a novel, synergistic therapeutic avenue to combat this formidable disease.

## 2 Methods

### 2.1 Protocol design and registration

This systematic review was conducted in accordance with the Cochrane Handbook and PRISMA guidelines (12). The study protocol has been registered and approved on PROSPERO. (registration no. CRD420251229183).

### 2.2 Literature search strategy

Databases Searched: Computerized searches were performed across the following six databases: PubMed, Web of Science, Embase, Cochrane Library, Scopus, and CNKI. The search period spanned from the inception of each database until April 8, 2026. Search keywords and strategies were formulated based on the PICOS framework and Medical Subject Headings (MeSH). The detailed search strategies are provided in the Supplementary materials.

### 2.3 Inclusion and exclusion criteria

#### 2.3.1 Inclusion criteria

P (Population): Mouse models.

I (Intervention): Electroacupuncture alone.

C (Comparator): Untreated ALS-SOD1^G93A^ transgenic models.

O (Outcomes): The primary outcome was motor coordination performance assessed by the rotarod test. Secondary outcomes comprised the following domains: (1) general health status: body weight, disease onset time, and survival period; (2) behavioral assessments: open field test; (3) neuroinflammatory markers: ionized calcium-binding adapter molecule 1 (Iba-1), tumor necrosis factor-alpha (TNF-*α*), interleukin-6 (IL-6), interleukin-1 beta (IL-1β), Toll-like receptor 4 (TLR4), nuclear factor-kappa B (NF-κB), p38 mitogen-activated protein kinase (p38), and phosphorylated p38 (p-p38); (4) apoptosis-related factors: phosphorylated protein kinase B (p-AKT) and phosphorylated glycogen synthase kinase-3 beta (p-GSK-3β); (5) regulation of RNA homeostasis: TAR DNA-binding protein 43 (TDP-43); (6) inhibition of axonal regeneration impairment: Ras homolog gene family member A (RhoA).

S (Study design): All experimental study design investigating the effects of electroacupuncture on ALS mouse models.

#### 2.3.2 Exclusion criteria

(1) Studies employing combined therapies (e.g., electroacupuncture plus pharmacotherapy) rather than electroacupuncture alone.
(2) Case reports, clinical trials, crossover studies, reviews, meta-analyses, dissertations/theses, or conference abstracts.
(3) Studies not focused on amyotrophic lateral sclerosis or not involving animal experiments.
(4) Duplicate publications.
(5) Studies with incomplete data or of poor methodological quality.
(6) Studies that cannot be translated.

### 2.4 Data extraction

Two investigators independently performed literature screening and data extraction. The results were cross-checked, and any discrepancies were resolved through discussion with a third investigator to reach a consensus. Literature screening was conducted using EndNote (version X9), and data extraction was performed with GetData Graph Digitizer (version 2.26). All assessments were carried out independently, and the final data for inclusion were determined by comparing the results.

Prior to formal data extraction, a preliminary data extraction form was developed based on the study objectives and inclusion criteria. The extracted parameters included: study characteristics (author, publication year, country, model type); electroacupuncture protocol (acupoint selection, stimulation frequency, treatment duration); and outcome measures (general condition/behavioral tests/neuroinflammatory factors/cell survival and apoptosis factors / neuronal homeostasis disruptors/neuron count). To validate the effectiveness and feasibility of the form, a pilot test was conducted using approximately 10% of the initially identified potentially eligible studies.

### 2.5 Quality assessment

Risk of bias assessment was conducted using SYRCLE’s ROB tool (13), with ten domains evaluated as low, unclear, or high risk. Draw a summary chart of bias risk using Review Manager 5.4.1. Mean scores were calculated to quantify overall bias risk, ensuring methodological robustness. Methodological quality was assessed using the CAMARADES checklist (14), with studies evaluated across ten criteria. Mean scores were computed to quantify study quality. Discrepancies were adjudicated through consensus with a third investigator.

Detailed assessment criteria are provided in the Supplementary materials.

### 2.6 Data analysis

Statistical analysis was conducted using Stata 15 and R software. Stata 15 served as the primary tool for analyses including generating forest plots, performing sensitivity analyses, creating funnel plots, and conducting subgroup analyses. R software was employed for subsequent statistical computations and graphical refinements. For continuous outcome variables, the mean difference (MD) or standardized mean difference (SMD) was used as the effect measure. Heterogeneity was quantified using the *I*^2^ statistic. A fixed-effects model was applied to assess heterogeneity if the included studies were homogeneous in terms of animal model, age, sex, and housing conditions, or if *I*^2^ ≤ 50%. Otherwise, a random-effects model (inverse variance method) was used. Substantial heterogeneity was addressed through sensitivity analysis, which involved sequentially excluding individual studies to identify potential sources of variation. Residual heterogeneity was further explored via stratified subgroup analysis and meta-regression. For primary outcome measures with 10 or more included studies, publication bias was assessed using Egger’s test, Begg’s test, funnel plot asymmetry, and the trim-and-fill method.

## 3 Results

### 3.1 Literature screening process

**Figure 1:**
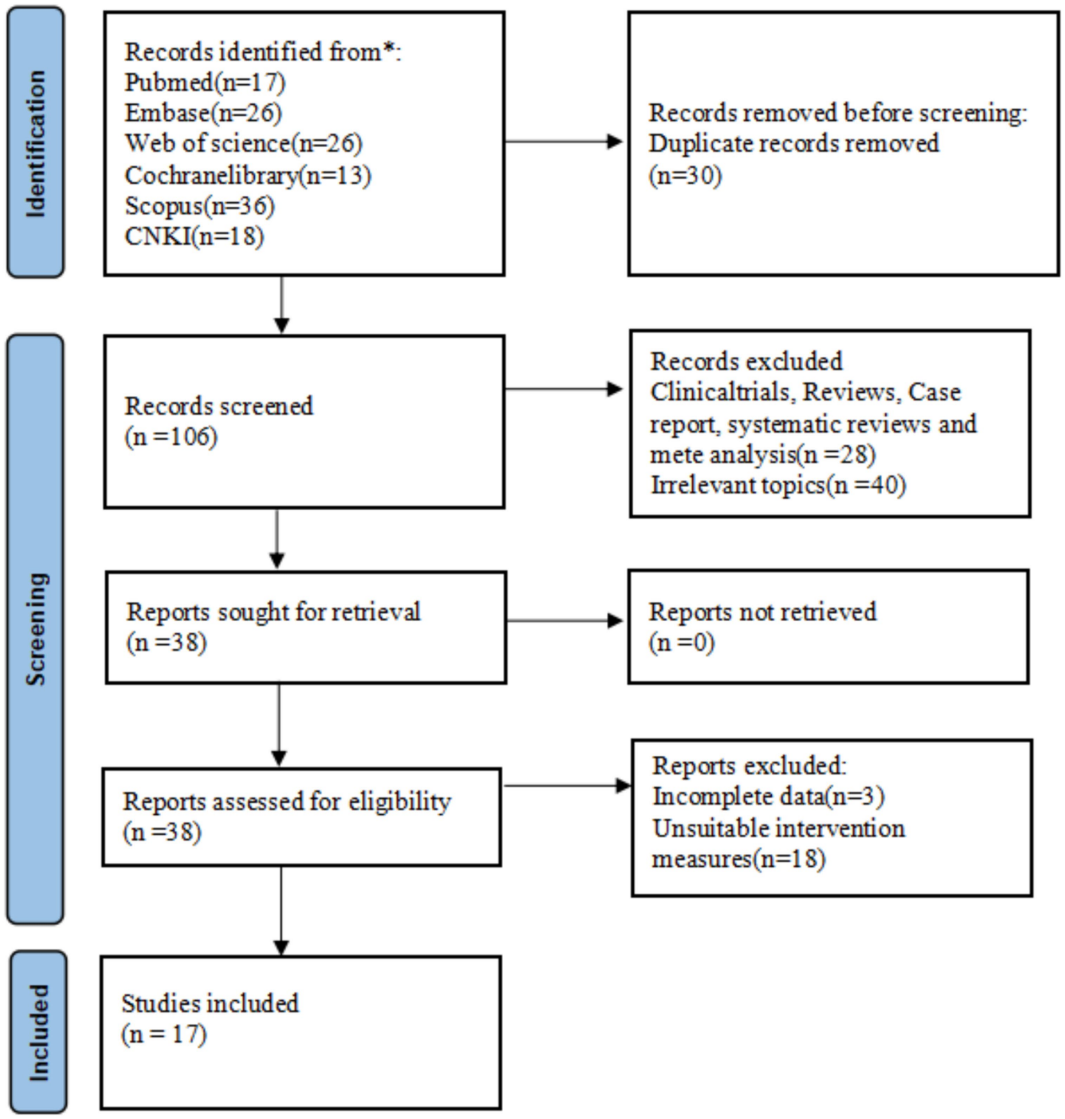
Literature search and screening process.

Using search strategies formulated based on MeSH terms, 136potentially relevant records were retrieved from database inception to April 8, 2026. After removing 30 duplicate records, the titles and abstracts of the remaining 106 records were screened. A total of 68 studies were excluded based on titles, among which 28 studies were clinical trials, reviews, case reports, systematic reviews, and meta-analyses, and the other 40 studies were irrelevant topics. After reading the abstracts and full texts, 3 studies were excluded due to incomplete data, and 18 studies were excluded because the interventions did not meet the inclusion criteria. Ultimately, 17 studies (15–31) were included in the analysis. The basic characteristics of the included studies were summarized, encompassing: author and publication year, country, animal model, sample size, electroacupuncture acupoint, stimulation frequency, treatment duration, waveform type, outcome measure, and quality assessment score. The literature search and selection process is illustrated in Figure 1, and the detailed characteristics of the included studies are presented in Table 1.

**Table 1:** List of research information.

Author (year)	Country	Animal model	Acupuncture measure						Outcome measure	Score	Cite
			*N*	Acupuncture location	Frequency	Time	Cot	Waveform			
HuaJiang (2011)	Korea	SOD1^G93A^	20	ST36	2/w	30 min	110d	Continuous Wave	⑥⑦⑧⑩⑮	5	(15)
Yang (2010)	Korea	SOD1^G93A^	28	ST36	2/w	30 min	110d	Continuous Wave	④⑥⑦	5	(16)
Zhao (2025)	China	SOD1^G93A^	16	GV20, BL10, ST25	5/w	10 min	3w	Continuous Wave	⑤⑥⑧⑨	8	(17)
Liu (2025)	China	SOD1^G93A^	24	GV20, BL10, ST25	5/w	10 min	5w	Continuous Wave	④⑤⑥⑦⑨⑯⑰	6	(18)
Sun (2021)	China	SOD1^G93A^	24	ST36, LI11	5/w	15 min	4w	Intermittent Wave	①④	6	(19)
Lu (2024)	China	SOD1^G93A^	30	GV20, BL10, ST25	5/w	10 min	4w	Continuous Wave	④⑥⑦⑯⑰	7	(20)
Wang (2022)	China	SOD1^G93A^	24	EX-B2	2/w	20 min	4w	Continuous Wave	②④⑨⑩⑪⑱	8	(21)
Sun (2020)	China	SOD1^G93A^	24	EX-B2	2/w	20 min	4w	Continuous Wave	⑭⑮	8	(22)
Sun (2021)	China	SOD1^G93A^	24	EX-B2	2/w	20 min	4w	Continuous Wave	④	8	(23)
Su (2019)	China	SOD1^G93A^	30	EX-B2	2/w	20 min	4w	Continuous Wave	⑦⑧⑨⑫⑬	7	(24)
Sun (2016)	China	SOD1^G93A^	20	EX-B2	2/w	20 min	4w	Continuous Wave	①②③④	6	(25)
Su (2019)	China	SOD1^G93A^	12	EX-B2	2/w	20 min	4w	Continuous Wave	④⑭	7	(26)
Guo (2017)	China	SOD1^G93A^	16	EX-B2	2/w	20 min	4w	Sparse wave	⑱	7	(27)
Wei (2017)	China	SOD1^G93A^	12	EX-B2	2/w	20 min	4w	Continuous Wave	⑫⑬	7	(28)
Liu (2023)	China	SOD1^G93A^	20	ST36, GB34	5/w	20 min	2w	Continuous Wave	④⑤⑩⑰	8	(29)
Liu (2021)	China	SOD1^G93A^	12	ST36, SP6, LI11, LI4	5/w	30 min	8w	Intermittent Wave	①②③	8	(30)
Wang (2023)	China	SOD1^G93A^	36	EX-B2	2/w	20 min	4w	Continuous Wave	②③④⑥⑦⑩⑪	8	(31)

①Body weight ② Disease onset time ③ Survival period ④ Rotarod test ⑤ Open field test⑥Iba-1⑦TNF-α⑧IL-6⑨IL-1β⑩NF-κB⑪TLR4⑫p38 ⑬p-p38.

⑭p-GSK-3β⑮p-AKT⑯RhoA⑰TDP-43⑱Motor Neuron Count.

N: sample size; Cot, treatment duration; w, week; d, Day; Score: Quality Scores of Included Studies.

### 3.2 Study quality assessment

**Figure 2:**
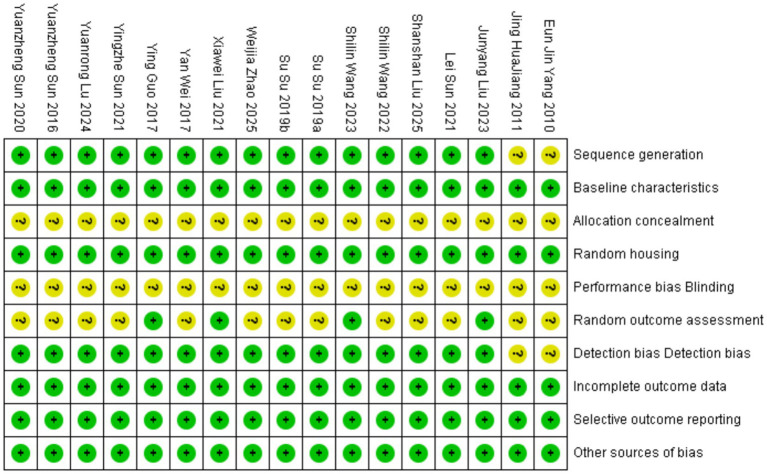
Risk of Bias analysis.

**Figure 3:**
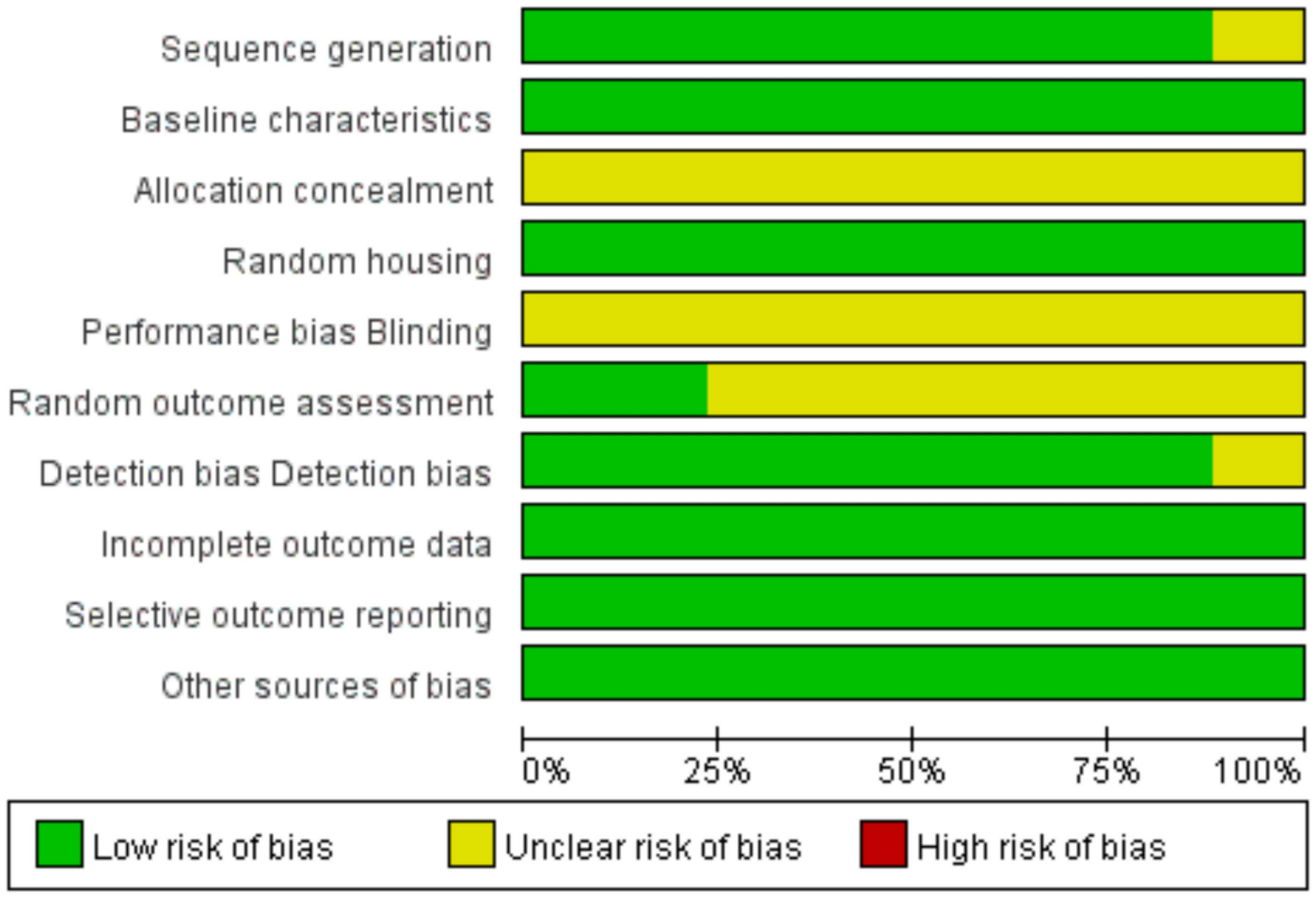
Risk of Bias Summary.

The risk of bias in the 17 included studies was assessed using the SYRCLE’s Risk of Bias (ROB) tool. The detailed assessment results are presented in Figures 2, 3 and Supplementary materials. The quality of the 17 included studies was evaluated using the CAMARADES checklist. According to the scoring standards, the scores ranged from 5 to 8, with an average score of 7. The detailed quality assessment is shown in Table 2.

**Table 2:** Quality assessment of included studies.

Author (Year)	1	2	3	4	5	6	7	8	9	10	Score	Cite
Jiang (2011)	Y	Y	Y	?	?	Y	Y	?	?	?	5	(15)
Yang (2010)	Y	Y	Y	?	?	Y	Y	?	?	?	5	(16)
Zhao (2025)	Y	Y	Y	Y	Y	Y	Y	?	Y	?	8	(17)
Liu (2025)	Y	Y	Y	?	Y	Y	Y	?	Y	?	6	(18)
Sun (2021)	Y	Y	Y	?	Y	Y	Y	?	Y	?	6	(19)
Lu (2024)	Y	Y	Y	?	Y	Y	Y	?	Y	?	7	(20)
Wang (2022)	Y	Y	Y	Y	Y	Y	Y	?	Y	?	8	(21)
Sun (2020)	Y	Y	Y	Y	Y	Y	Y	?	Y	?	8	(22)
Sun (2021)	Y	Y	Y	Y	Y	Y	Y	?	Y	?	8	(23)
Su (2019)	Y	Y	Y	Y	Y	Y	Y	?	?	?	7	(24)
Sun (2016)	Y	?	Y	Y	Y	Y	Y	?	?	?	6	(25)
Su (2019)	Y	Y	Y	Y	Y	Y	Y	?	?	?	7	(26)
Guo (2017)	Y	Y	Y	Y	Y	Y	Y	?	?	?	7	(27)
Wei (2017)	Y	Y	Y	Y	Y	Y	Y	?	?	?	7	(28)
Liu (2023)	Y	Y	Y	Y	Y	Y	Y	?	Y	?	8	(29)
Liu (2021)	Y	Y	Y	Y	Y	Y	Y	?	Y	?	8	(30)
Wang (2023)	Y	Y	Y	Y	Y	Y	Y	?	Y	?	8	(31)

Y, yes; N, no;?, unclear.

### 3.3 Meta-analysis results

#### 3.3.1 General conditions

##### 3.3.1.1 Body weight

**Figure 4:**
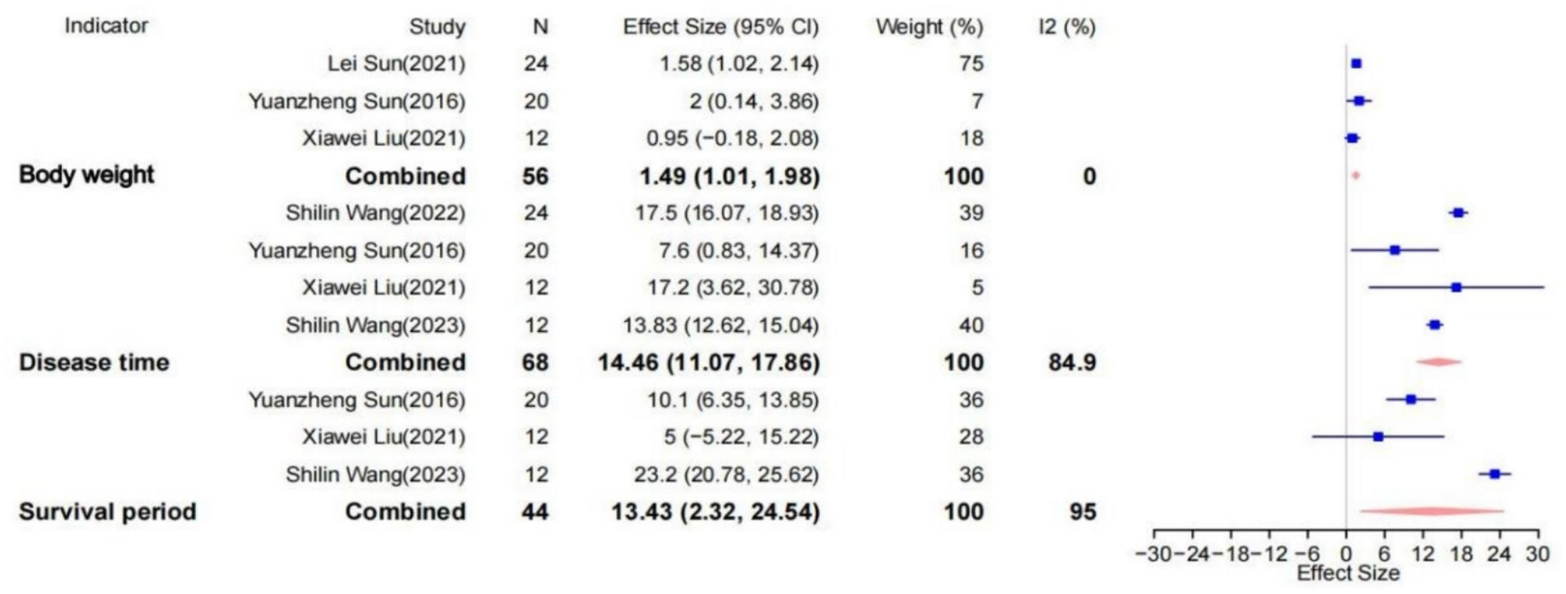
General conditions forest plot.

Three studies reported on body weight(g), involving a total of 56 animal subjects. The heterogeneity test indicated low heterogeneity (*I^2^* = 0%, *p* = 0.534), therefore a fixed-effects model was applied. The pooled analysis showed a significant effect [MD = 1.49, 95% CI (1.01, 1.98), *Z* = 6.010, *p* < 0.001] (Figure 4).

##### 3.3.1.2 Disease time

Four studies reported on disease onset time (day), involving a total of 68 animal subjects. Significant heterogeneity was observed (*I*^2^ = 84.9%, *p* < 0.001), leading to the use of a random-effects model. The pooled analysis demonstrated a significant effect [MD = 14.46, 95% CI (11.07, 17.86), *Z* = 8.352, *p* < 0.001] (Figure 4).

##### 3.3.1.3 Survival period

Three studies reported on the survival period(day), involving a total of 44 animal subjects. Significant heterogeneity was present (*I*^2^ = 95.0%, *p* < 0.001), warranting a random-effects model. The pooled analysis revealed a significant effect [MD = 13.43, 95% CI (2.32, 24.54), *Z* = 2.368, *p* = 0.018] (Figure 4).

#### 3.3.2 Behavioral indicators

##### 3.3.2.1 Rotarod test

**Figure 5:**
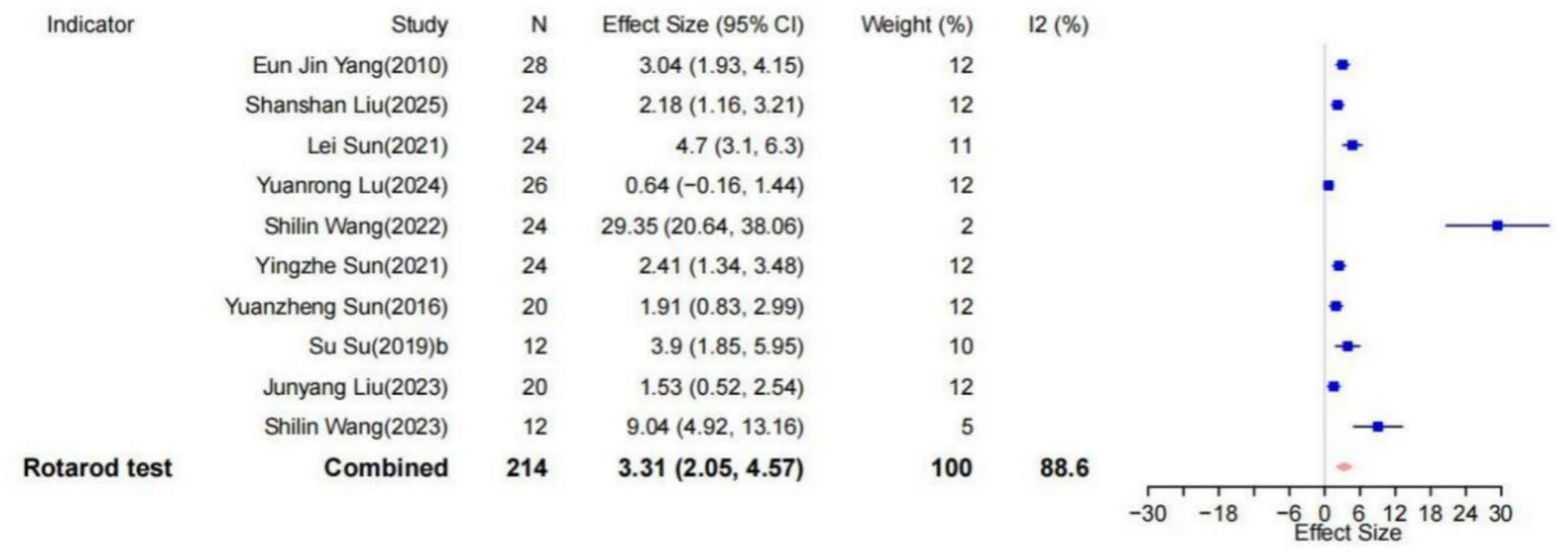
Rotarod test forest plot.

Ten studies reported the rotarod test results, involving a total of 214 animals. The heterogeneity test indicated high heterogeneity (*I*^2^ = 88.6%, *p* < 0.001), therefore a random-effects model was applied. The pooled analysis demonstrated a statistically significant effect [SMD = 3.31, 95% CI (2.05, 4.57), *Z* = 5.151, *p* < 0.001], as shown in Figure 5.

##### 3.3.2.2 Open field test

**Figure 6:**
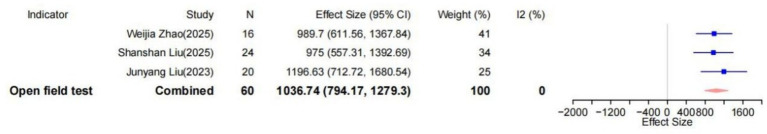
Open field test forest plot.

Three studies reported open field test(cm), results, involving a total of 56 animals. Low heterogeneity was observed (*I^2^* = 0%, *p* = 0.755), and thus a fixed-effects model was used. The pooled analysis showed a statistically significant effect [MD = 1036.74, 95% CI (794.17, 1279.30), *Z* = 8.377, *p* < 0.001], as presented in Figure 6.

#### 3.3.3 Neuroinflammation

##### 3.3.3.1 Iba-1

**Figure 7:**
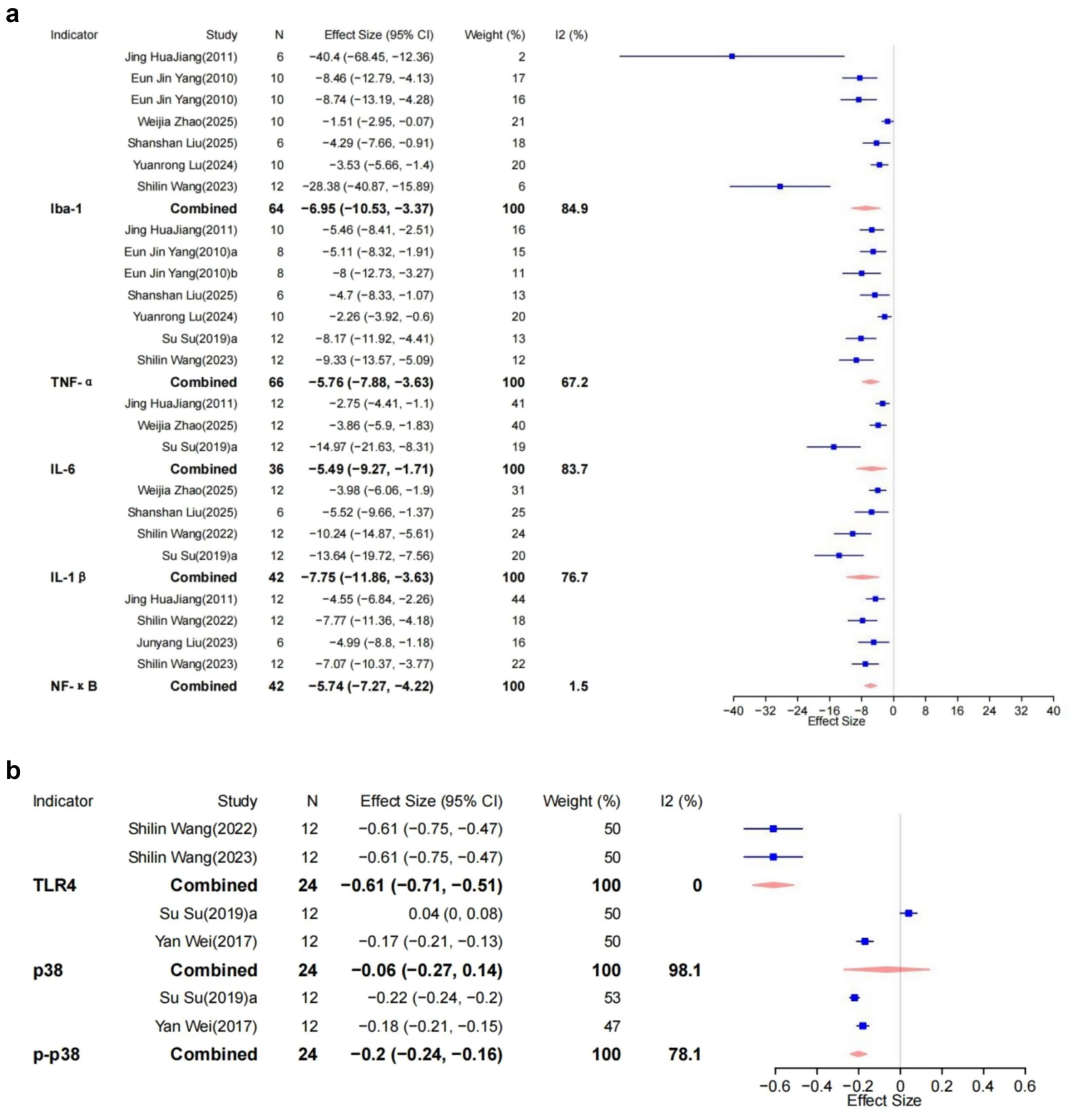
**(A, B)** Neuroinflammation forest plot.

Six studies reported Iba-1 levels, involving a total of 64 animals. High heterogeneity was detected (*I*^2^ = 84.9%, *p* < 0.001), leading to the application of a random-effects model. The pooled analysis revealed a statistically significant effect [SMD = −6.95, 95% CI (−10.53, −3.37), *Z* = −3.808, *p* < 0.001], as shown in Figure 7.

##### 3.3.3.2 TNF-*α*

Six studies reported TNF-α levels, involving a total of 66 animals. High heterogeneity was observed (*I*^2^ = 67.2%, *p* = 0.006), and a random-effects model was used. The pooled analysis demonstrated a statistically significant effect [SMD = −5.76, 95% CI (−7.88, −3.63), *Z* = −5.309, *p* < 0.001] (Figure 7).

##### 3.3.3.3 Il-6

Three studies reported IL-6 levels, involving a total of 36 animals. Significant heterogeneity was present (*I*^2^ = 83.7%, *p* = 0.002), warranting a random-effects model. The pooled analysis indicated a statistically significant effect [SMD = −5.49, 95% CI (−9.27, −1.71), *Z* = −2.847, *p* = 0.004] (Figure 7).

##### 3.3.3.4 Il-1β

Four studies reported IL-1β levels, involving a total of 42 animals. High heterogeneity was found (*I*^2^ = 76.7%, *p* = 0.005), and a random-effects model was applied. The pooled analysis showed a statistically significant effect [SMD = −7.75, 95% CI (−11.86, −3.63), *Z* = −3.688, *p* < 0.001] (Figure 7).

##### 3.3.3.5 NF-κB

Four studies reported NF-κB levels, involving a total of 42 animals. Low heterogeneity was observed (*I*^2^ = 1.5%, *p* = 0.385), therefore a fixed-effects model was used. The pooled analysis demonstrated a statistically significant effect [SMD = −5.74, 95% CI (−7.27, −4.22), *Z* = −7.731, *p* < 0.001] (Figure 7).

##### 3.3.3.6 TLR4

Two studies reported TLR4 levels, involving a total of 24 animals. The heterogeneity test indicated no heterogeneity (*I*^2^ = 0%, *p* = 1.000), therefore a fixed-effects model was applied. The pooled analysis revealed a statistically significant effect [MD = −0.61, 95% CI (−0.71, −0.51), *Z* = −12.100, *p* < 0.001] (Figure 7).

##### 3.3.3.7 p38 and p-p38

Two studies reported p38 and p-p38 levels, involving a total of 24 animals. For p38, significant heterogeneity was observed (*I*^2^ = 98.1%, *p* < 0.001), and a random-effects model was used. The pooled analysis showed no statistically significant effect [MD = −0.06, 95% CI (−0.27, 0.14), *Z* = −0.619, *p* = 0.536]. For p-p38, high heterogeneity was detected (*I*^2^ = 78.1%, *p* = 0.033), and a random-effects model was applied. The pooled analysis demonstrated a statistically significant effect [MD = −0.20, 95% CI (−0.24, −0.16), *Z* = −10.066, *p* < 0.001] (Figure 7).

#### 3.3.4 Inhibition of apoptosis

##### 3.3.4.1 P-GSK-3β

**Figure 8:**
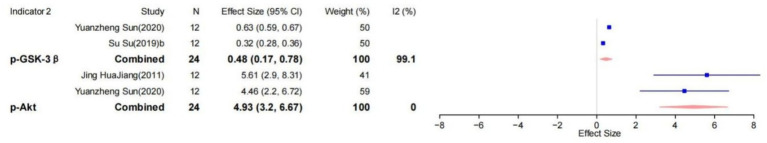
Inhibition of apoptosis forest plot.

Two studies reported p-GSK-3β levels, involving a total of 24 animals. Significant heterogeneity was observed (*I^2^* = 99.1%, *p* < 0.001), and a random-effects model was used. The pooled analysis demonstrated a statistically significant effect [MD = 0.48, 95% CI (0.17, 0.78), *Z* = 3.064, *p* = 0.002] (Figure 8).

##### 3.3.4.2 P-Akt

Two studies reported p-Akt levels, involving a total of 24 animals. The heterogeneity test indicated low heterogeneity (*I*^2^ = 0%, *p* = 0.525), therefore a fixed-effects model was applied. The pooled analysis revealed a statistically significant effect [SMD = 4.93, 95% CI (3.20, 6.67), *Z* = 5.575, *p* < 0.001] (Figure 8).

#### 3.3.5 Inhibition of axonal regeneration impairment

##### 3.3.5.1 RhoA

**Figure 9:**
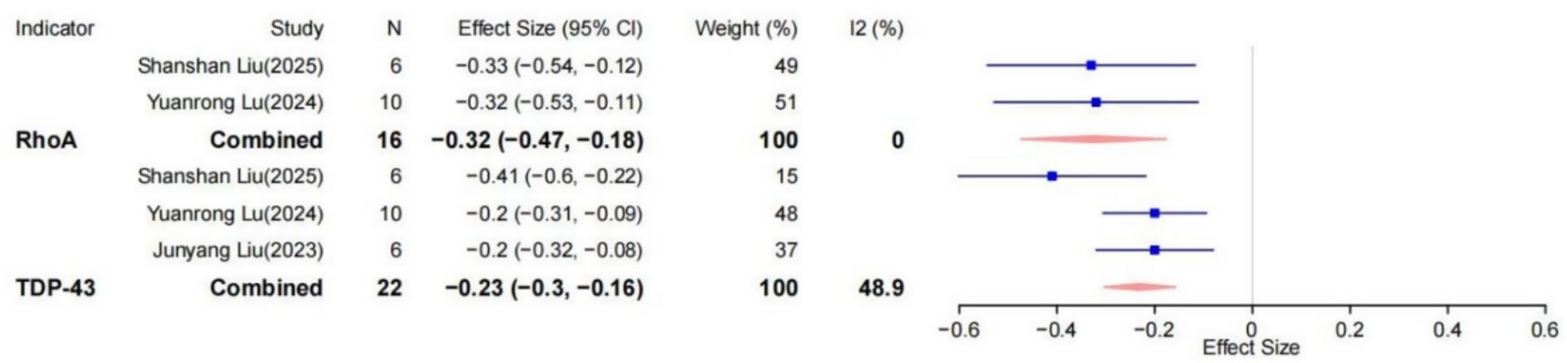
RNA homeostasis in axonal regeneration forest plot.

Two studies reported RhoA levels, involving a total of 16 animals. The heterogeneity test indicated no heterogeneity (*I*^2^ = 0%, *p* = 0.948), therefore a fixed-effects model was applied. The pooled analysis revealed a statistically significant effect [MD = −0.32, 95% CI (−0.47, −0.18), *Z* = −4.272, *p* < 0.001] (Figure 9).

#### 3.3.6 Regulation of RNA homeostasis

##### 3.3.6.1 TDP-43

Three studies reported TDP-43 levels, involving a total of 22 animals. Low heterogeneity was observed (*I*^2^ = 48.9%, *p* = 0.142), and a fixed-effects model was used. The pooled analysis demonstrated a statistically significant effect [MD = −0.23, 95% CI (−0.30, −0.16), *Z* = −6.153, *p* < 0.001] (Figure 9).

#### 3.3.7 Histopathological findings

##### 3.3.7.1 Motor neuron count

**Figure 10:**
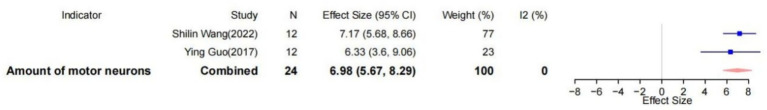
Motor neuron count.

Two studies reported the motor neuron count(amount), in the lumbar anterior horn, involving a total of 24 animals. The heterogeneity test indicated no heterogeneity (*I*^2^ = 0%, *p* = 0.597), therefore a fixed-effects model was applied. The pooled analysis demonstrated a statistically significant increase in the motor neuron count [MD = 6.98, 95% CI (5.67, 8.29), *Z* = 10.448, *p* < 0.001] (Figure 10).

### 3.4 Sensitivity and subgroup analyses

Substantial heterogeneity (*I*^2^ > 50%) was observed across multiple outcomes: Disease time, Survival period, Rotarod test, Iba-1, TNF-*α*, IL-6, IL-1β, p38, p-p38, p-GSK-3β. Random-effects models were applied to account for this variability. To verify the robustness of our findings, sensitivity analyses were conducted according to the bias risk levels of included studies. Sequential exclusion of individual studies demonstrated no significant alterations in outcome estimates, confirming high stability and reliability of the pooled results (Detailed information can be found in the Supplementary material).

Given the high heterogeneity observed in the primary outcome measure (Rotarod test), its sources were further explored via subgroup analysis and meta-regression. Studies were categorized into seven subgroups based on: country, acupoint location, stimulation frequency, session duration, treatment period, waveform, and quality score. The summary analyses indicated that none of these factors were significant sources of heterogeneity. Detailed subgroup analysis results are provided in the Supplementary materials. The meta-regression results are presented in Table 3.

**Table 3:** Meta-regression results.

Outcomes	Variable	*p*-value
Rotarod test	Country	0.438
	Acupuncture location	0.110
	Frequency	0.136
	Time	0.892
	Cot	0.424
	Waveform	0.567
	Scores	0.108

### 3.5 Assessment of publication Bias

**Figure 11:**
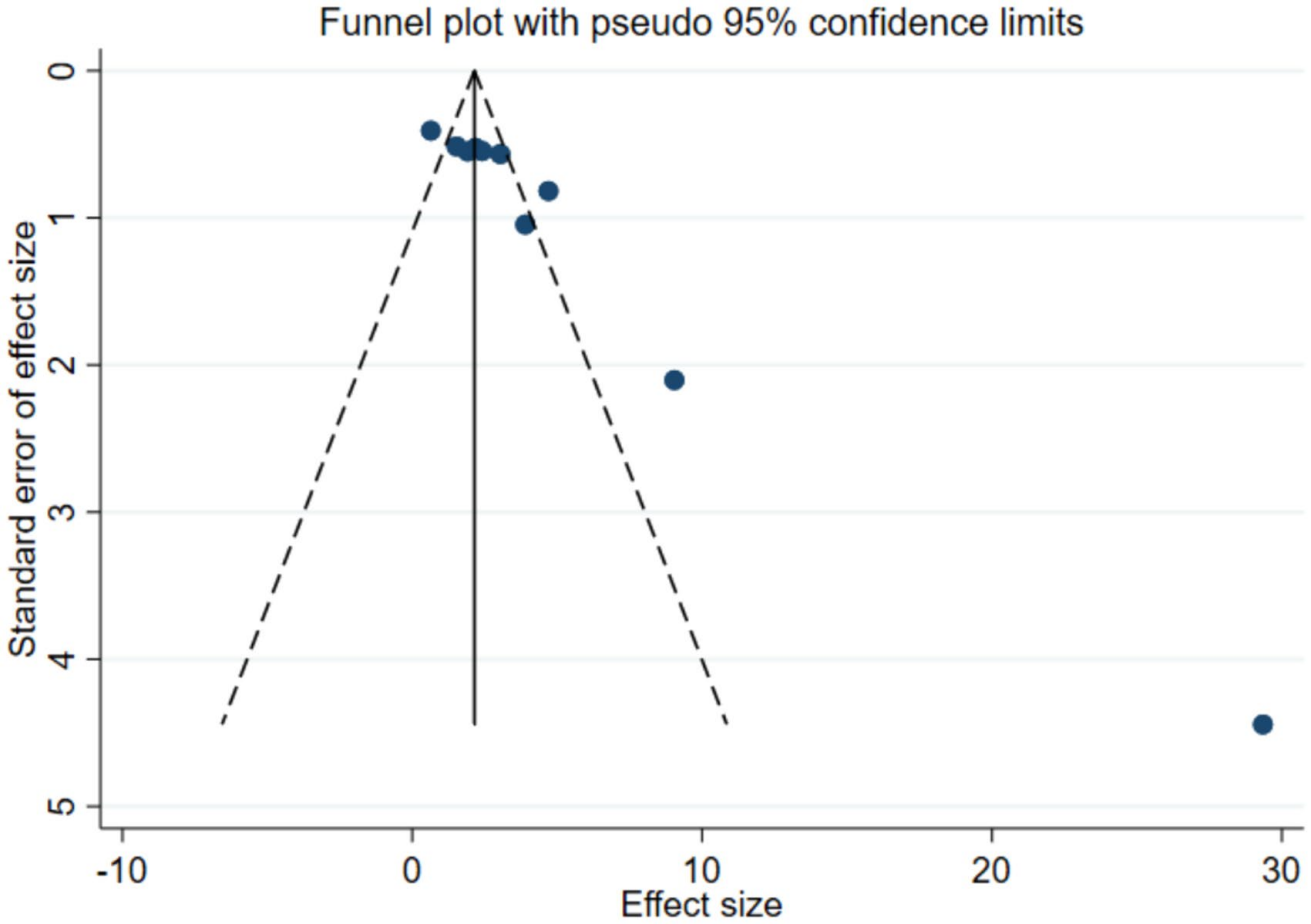
Funnel plot of rotarod test.

**Figure 12:**
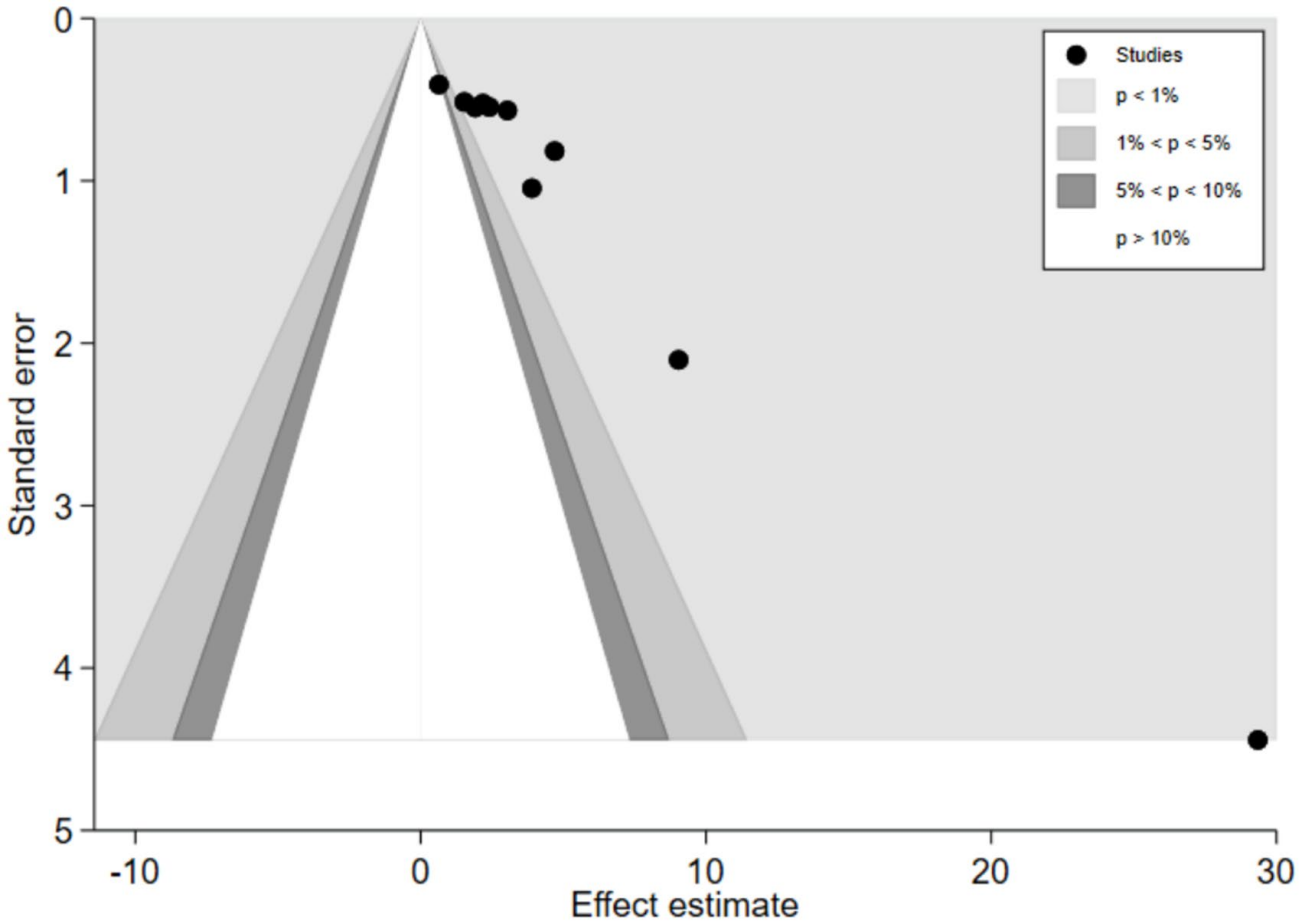
Contour-enhanced funnel plot of rotarod test.

**Figure 13:**
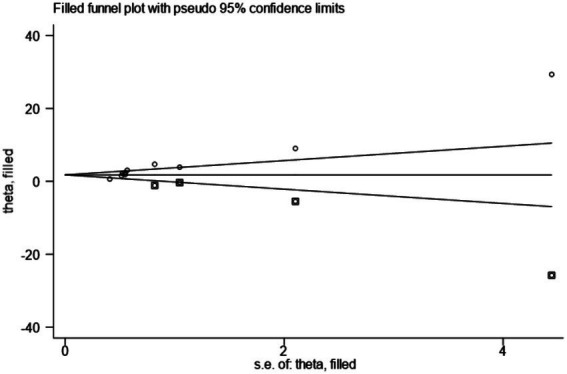
Trim-and-fill analysis of rotarod test.

Publication bias assessment for the 10 studies reporting Rotarod test scores indicated potential bias, as suggested by an asymmetrical funnel plot distribution (*t* = 7.33, *p* < 0.001) (Figure 11). Further analysis using contour-enhanced funnel plots and the trim-and-fill method (Figures 12, 13) confirmed the presence of significant publication bias (*p* < 0.001).

## 4 Discussion

### 4.1 Electroacupuncture exerts therapeutic effects on ALS through multi-target modulation

This study comprehensively evaluated the multi-dimensional therapeutic effects of EA in SOD1^G93A^ transgenic ALS mouse models. As a progressive neurodegenerative disease, the pathological progression of ALS involves complex interactions within molecular networks. Our results demonstrate that EA intervention not only significantly improved the overall health status and behavioral performance of the animals but also exerted multi-target therapeutic effects, thereby delaying disease progression and extending survival.

In chronic debilitating neurodegenerative diseases like ALS, the maintenance of body weight serves as a crucial indicator of general nutritional status and disease burden (32). Disease onset time and survival period are ultimate indicators for assessing the rate of disease progression and the final efficacy of therapeutic interventions (33). This study found that EA treatment significantly improved the general health status of ALS model mice, specifically manifesting as a slowed decline in body weight, a significantly delayed disease onset time (*p* < 0.001), and an extended survival period (*p* < 0.05) compared to the model group. These results suggest that EA intervention positively delays the overall disease course, establishing a phenotypic foundation for further investigation into its neuroprotective mechanisms. For the behavioral assessment of motor function, the rotarod test and the open field test were employed to evaluate motor coordination/endurance and autonomous exploratory activity, respectively. Deficits in these behaviors directly correspond to the progressive muscle weakness and loss of motor function experienced by ALS patients (34, 35). Our observations revealed that mice in the EA treatment group exhibited significantly prolonged latency to fall in the rotarod test and increased total movement distance in the open field test (*p* < 0.001). This indicates that EA not only improved the animals’ basic motor capacity but may also have alleviated anxiety-like states and enhanced central drive. The simultaneous improvement in these behavioral parameters suggests that EA treatment helps maintain the overall functionality of the motor system, providing a phenotypic basis for subsequent exploration of molecular mechanisms.

Neuroinflammation is a core pathological mechanism driving ALS disease progression (36). Microglia, as the primary immune cells of the central nervous system, exhibit upregulated expression of the activation marker Iba-1, signaling the initiation of neuroinflammation (37). Upon sensing danger signals through receptors like TLR4, microglia activate key signaling pathways such as NF-κB, subsequently promoting the transcription and release of pro-inflammatory cytokines including TNF-*α*, IL-6, and IL-1β (38, 39). The phosphorylation of p38 (p-p38), a key kinase in inflammatory signaling, further amplifies the inflammatory response. These inflammatory mediators collectively create a toxic environment that directly damages motor neurons and accelerates their death. This study confirmed that EA significantly reduced the protein expression levels of Iba-1, TLR4, NF-κB, and p-p38 (*p* < 0.001) and downregulated the levels of TNF-α, IL-6, and IL-1β (*p* < 0.01). These findings indicate that EA attenuates the microglia- mediated neuroinflammatory axis, ultimately reducing the storm of downstream pro-inflammatory factors and fostering a more favorable survival microenvironment for motor neurons.

Regarding the regulation of cell survival and apoptosis, the phosphoinositide 3-kinase (PI3K)/protein kinase B (Akt) signaling pathway is one of the most crucial pro-survival pathways within cells. Akt is a central pro-survival kinase, and its p-Akt transmits potent anti-apoptotic signals downstream. GSK-3β is a key downstream target of Akt; it possesses inherent pro-apoptotic activity. However, Akt-mediated p-GSK-3β inhibits its activity, thereby exerting neuroprotective effects (40). In ALS, the function of this pro-survival pathway is often compromised (41). The present study found that EA intervention significantly increased the protein levels of p-Akt and p-GSK-3β in the lumbar spinal cord tissue of ALS mice (*p* < 0.01). This discovery suggests that the neuroprotective effect of EA may be partly achieved by activating the PI3K/Akt signaling pathway, which in turn inhibits the activity of the pro-apoptotic protein GSK-3β. This mechanism provides robust anti-apoptotic protection for motor neurons, thereby slowing their loss.

Axonal regeneration impairment is a significant factor contributing to neuromuscular junction denervation and functional irrecoverability in ALS (42). RhoA serves as a key negative intrinsic regulator of axonal growth. Upon activation by upstream inhibitory signals, RhoA triggers downstream cascades that lead to actin cytoskeleton contraction and growth cone collapse, thereby potently inhibiting axonal regeneration and remodeling (43). In ALS, the persistent activation of the RhoA pathway forms a crucial molecular basis hindering neuromuscular junction repair and collateral sprouting (44). This study found thatEA treatment effectively reduced RhoA activity (*p* < 0.001). This suggests that EA may, by alleviating the RhoA-mediated inhibition of axonal growth, partially liberate the intrinsic repair capacity of motor neurons and improve axonal structural plasticity, which is vital for maintaining neuromuscular junction function and delaying paralysis.

Regarding RNA function regulation, the normal role of TDP-43 involves regulating RNA metabolism, including splicing, transport, and stability. Its abnormal aggregation not only results in the loss of normal RNA regulatory function but also confers a gain-of-function toxicity, leading to widespread RNA metabolic disturbances and proteostasis imbalance (45). Aberrant cytoplasmic aggregation of TDP-43 represents a hallmark molecular pathological event in the vast majority of ALS cases (46). In this study, we observed that EA treatment alleviated the pathological aggregation of TDP-43 in the cytoplasm (*p* < 0.001). This implies that EA may mitigate the consequent RNA metabolic crisis and direct neurotoxicity by either maintaining the normal nuclear localization and function of TDP-43 or promoting the clearance of its abnormal aggregates, which could be a pivotal link in its mechanism of action.

The improvements observed at the molecular and behavioral levels were ultimately corroborated by definitive histopathological evidence. The progressive loss of motor neurons in the anterior horn of the lumbar spinal cord represents the terminal manifestation of ALS pathology, directly leading to paralysis (47). The results of this study showed that ALS model mice treated with EA had a significantly greater number of surviving motor neurons in the lumbar anterior horn compared to the model group (*p* < 0.001). The successful preservation of motor neurons constitutes the ultimate manifestation of EA’s neuroprotective function mediated through the aforementioned multi-target mechanisms and serves as the direct structural basis for its ability to delay muscle paralysis and extend survival. This finding provides morphological confirmation of EA’s neuroprotective efficacy.

In summary, EA does not act through a single pathway but establishes a synergistic therapeutic network. By modulating multiple interrelated core pathological processes—including neuroinflammation, cell survival and apoptosis, signals inhibiting axonal regeneration, and RNA metabolism—EA ultimately achieves effective protection of motor neurons. This protection is comprehensively reflected in the maintenance of body weight, improvement in behavioral performance, and extension of survival time. This work elucidates the biological foundation underlying the neuroprotective effects of EA in treating ALS, providing robust scientific evidence supporting EA as a promising adjunctive therapeutic strategy for ALS.

### 4.2 Comparison with existing research

Through a systematic meta-analysis, this study integrated well-designed preclinical animal studies to comprehensively investigate the multi-target mechanisms of EA intervention in ALS. The core findings encompass the modulation of several key pathological processes, including neuroinflammation, cell apoptosis, inhibition of axonal regeneration, and RNA metabolic disturbances. In contrast to prior meta-analyses that primarily summarized clinical efficacy, the present study implemented standardized quality control for the included animal experiments, thereby reducing heterogeneity across studies (48). This work is the first to systematically construct an interconnected network at the mechanistic level, encompassing inflammation, apoptosis, neurotrophic signaling, and axonal regeneration. This multidimensional integration of mechanisms addresses the previous lack of a systemic understanding of EA’s effects and establishes a solid theoretical foundation for positioning EA as a comprehensive management strategy for ALS, grounded in multi-target neuroprotective mechanisms.

### 4.3 Study limitations

First, despite conducting a thorough literature search, all 17 included articles originated from Asian countries. This phenomenon reflects a concentrated research interest in EA within Asia. It is important to note that this conclusion should be applied cautiously in other regions, such as Europe. Future research should strive to include multilingual and multinational literature to verify the global applicability of EA effects. Second, Substantial heterogeneity (*I*^2^ > 50%) was observed across multiple outcomes: Disease time, Survival period, Rotarod test, Iba-1, TNF-*α*, IL-6, IL-1β, p38, p-p38, p-GSK-3β, indicating high variability among the included studies. Publication bias was detected for the rotarod test, which may contribute to this heterogeneity. This publication bias could influence the study results, potentially leading to an overestimation of the actual effect of EA intervention. Although we attempted to estimate the impact of this bias using the trim-and-fill method and assessed the robustness of the findings through sensitivity analysis, these statistical approaches can only partially adjust for and evaluate the bias, not eliminate it entirely. Subgroup analysis and meta-regression did not identify any of the seven pre-specified factors as significant sources of heterogeneity. These findings highlight the necessity for enhanced methodological rigor in future research. The heterogeneity for the remaining outcomes might stem from variations in stimulation parameters (e.g., waveform), acupoint selection, intervention duration, or specific manipulation techniques. Finally, quality and risk-of-bias assessments identified key methodological limitations: (1) Issues in reporting randomization—no study described allocation concealment, and only three reported random outcome assessment. While a lack of reporting does not necessarily imply a lack of implementation, this omission significantly increases the risk of bias. Future animal studies should comprehensively report randomization procedures to enhance methodological transparency and result quality. (2) None of the included studies provided a conflict-of-interest statement, reflecting a common shortcoming. We strongly advocate for adherence to SYRCLE guidelines to mitigate potential bias. (3) No study reported a sample size calculation, indicating a lack of a fundamental experimental design step, which may hinder reproducibility. We strongly recommend that future studies incorporate a complete experimental design protocol to strengthen the credibility of their conclusions. (4) Animal welfare statements were absent in seven studies. While we believe these studies adhered to ethical standards, explicit statements are crucial for the completeness and ethical transparency of experimental research.

### 4.4 Clinical implications

The findings of this study hold clear implications for the clinical management of ALS. The multi-system regulatory properties demonstrated by EA suggest its potential for combination therapy with conventional drugs. A synergistic mechanism could enhance therapeutic efficacy or reduce the required dosage of single-agent treatments, thereby optimizing the current therapeutic window (49). He series of key molecular events identified (e.g., dynamics of specific inflammatory factors, changes in TDP-43 subcellular localization) lay the groundwork for developing quantifiable biomarkers of treatment response. This could support future patient stratification and personalized treatment planning within precision medicine practice. From a health economics perspective, EA is a highly accessible and relatively mature physical intervention modality (50). If its clinical value is validated through rigorous trials, EA could be integrated into multidisciplinary ALS management schemes. It could play roles at different disease stages, functioning to delay functional decline and improve quality of life, thereby alleviating the overall societal burden of this devastating disease.

## 5 Conclusion

The synthesized evidence from this systematic review and meta-analysis indicates that EA intervention exerts multi-target neuroprotective effects in experimental ALS models. Its mechanism of action involves the coordinated modulation of several interconnected pathological processes: suppressing neuroimmune overactivation (downregulating Iba-1, TLR4, NF-κB, p-p38, and related inflammatory factors), enhancing endogenous cell survival signaling (increasing p-Akt and p-GSK-3β levels), mitigating axonal regeneration inhibition (modulating RhoA activity), and improving nucleocytoplasmic proteostasis (reducing TDP-43 pathological aggregation). This synergistic action at the molecular level ultimately translates into the preservation of motor neuron counts, maintenance of neuromuscular function, and a significant extension of survival. To advance the clinical translation of this intervention strategy, there is an urgent need for methodologically rigorous randomized controlled trials to confirm its efficacy and safety in patient populations. A focused exploration of the quantitative relationship between molecular biomarkers and clinical outcome measures is also essential. This will provide decisive evidence for establishing an evidence-based, precise, and effective EA treatment paradigm.

## Data Availability

The original contributions presented in the study are included in the article/Supplementary material, further inquiries can be directed to the corresponding author.
