# Supplementary material for: Experimental evidence of electroacupuncture in ALS mouse models: a systematic review and meta-analysis
Source: Front Neurol. 2026 Jul 1;17:1780176. doi: 10.3389/fneur.2026.1780176 (PMC13368633; doi:10.3389/fneur.2026.1780176)
Supplement: Supplementary file 2 [file Table_2.DOC]

**Search Query**

| Database | Search strategy | Results |
| --- | --- | --- |
| 1) PubMed | #1 Acupuncture[Mesh] OR Acupuncture[Title/Abstract] OR Electroacupuncture[Title/Abstract] OR Electric acupuncture[Title/Abstract] OR Electro-acupuncture[Title/Abstract] OR Acupoint[Title/Abstract] | 45611 |
|  | #2 Amyotrophic Lateral Sclerosis[Mesh] OR Amyotrophic Lateral Sclerosis[Title/Abstract] OR Sclerosis, Amyotrophic Lateral[Title/Abstract] OR ALS[Title/Abstract] OR Gehrig's Disease[Title/Abstract] OR Gehrig Disease[Title/Abstract] OR Gehrigs Disease[Title/Abstract] OR Lou Gehrig Disease[Title/Abstract] OR Motor Neuron Disease, Amyotrophic Lateral Sclerosis[Title/Abstract] OR Lou Gehrig's Disease[Title/Abstract] OR Lou-Gehrigs Disease[Title/Abstract] OR Disease, Lou-Gehrigs[Title/Abstract] OR Charcot Disease[Title/Abstract] OR Amyotrophic Lateral Sclerosis With Dementia[Title/Abstract] OR Dementia With Amyotrophic Lateral Sclerosis[Title/Abstract] OR Amyotrophic Lateral Sclerosis, Guam Form[Title/Abstract] OR Guam Disease[Title/Abstract] OR Disease, Guam[Title/Abstract] OR Amyotrophic Lateral Sclerosis-Parkinsonism-Dementia Complex 1[Title/Abstract] OR Amyotrophic Lateral Sclerosis Parkinsonism Dementia Complex 1[Title/Abstract] OR Amyotrophic Lateral Sclerosis, Parkinsonism-Dementia Complex of Guam[Title/Abstract] OR Amyotrophic Lateral Sclerosis, Parkinsonism Dementia Complex of Guam[Title/Abstract] OR Guam Form of Amyotrophic Lateral Sclerosis[Title/Abstract] | 54477 |
|  | #3 Rat[Title/Abstract] OR Mice[Title/Abstract] OR Mouse[Title/Abstract] OR Animal[Title/Abstract] | 3075291 |
|  | #4 #1 AND #2 AND #3 | 17 |
| 2) Embase | #1 'acupuncture':ti,ab,kw OR 'electroacupuncture':ti,ab,kw OR 'electric acupuncture':ti,ab,kw OR 'electro-acupuncture':ti,ab,kw OR 'acupoint':ti,ab,kw | 56211 |
|  | #2 'amyotrophic lateral sclerosis':ti,ab,kw OR 'als':ti,ab,kw OR 'gehrig's disease':ti,ab,kw OR 'gehrig disease':ti,ab,kw OR 'gehrigs disease':ti,ab,kw OR 'lou gehrig disease':ti,ab,kw OR 'lou gehrig's disease':ti,ab,kw OR 'lou-gehrigs disease':ti,ab,kw OR 'charcot disease':ti,ab,kw OR 'amyotrophic lateral sclerosis with dementia':ti,ab,kw OR 'dementia with amyotrophic lateral sclerosis':ti,ab,kw OR 'guam disease':ti,ab,kw OR 'amyotrophic lateral sclerosis-parkinsonism-dementia complex 1':ti,ab,kw OR 'amyotrophic lateral sclerosis parkinsonism dementia complex 1':ti,ab,kw OR 'guam form of amyotrophic lateral sclerosis':ti,ab,kw | 72316 |
|  | #3 'rat':ti,ab,kw OR 'mice':ti,ab,kw OR 'mouse':ti,ab,kw OR 'animal':ti,ab,kw OR 'ROB':ti,ab,kw | 3942769 |
|  | #4 #1 AND #2 AND #3 | 26 |
| 3) Cochrane Library | #1 MeSH descriptor: [Acupuncture] explode all trees OR (Acupuncture OR Electroacupuncture OR Electric acupuncture OR Electro-acupuncture OR Acupoint):ti,ab,kw | 26872 |
|  | #2 MeSH descriptor: [Amyotrophic Lateral Sclerosis] explode all trees OR (Amyotrophic Lateral Sclerosis OR ALS OR Gehrig's Disease OR Gehrig Disease OR Gehrigs Disease OR Lou Gehrig Disease OR Lou Gehrig's Disease OR Lou-Gehrigs Disease OR Charcot Disease OR Amyotrophic Lateral Sclerosis With Dementia OR Dementia With Amyotrophic Lateral Sclerosis OR Guam Disease OR Amyotrophic Lateral Sclerosis-Parkinsonism-Dementia Complex 1 OR Amyotrophic Lateral Sclerosis Parkinsonism Dementia Complex 1 OR Guam Form of Amyotrophic Lateral Sclerosis):ti,ab,kw | 50768 |
|  | #3 (Rat OR Mice OR Mouse OR Animal):ti,ab,kw | 45931 |
|  | #4 #1 AND #2 AND #3 | 13 |
| 4) Web of science | #1 TS= (Acupuncture OR Electroacupuncture OR Electric acupuncture OR Electro-acupuncture OR Acupoint) | 36107 |
|  | #2 TS= (Amyotrophic Lateral Sclerosis OR ALS OR Gehrig's Disease OR Gehrig Disease OR Gehrigs Disease OR Lou Gehrig Disease OR Lou Gehrig's Disease OR Lou-Gehrigs Disease OR Charcot Disease OR Amyotrophic Lateral Sclerosis With Dementia OR Dementia With Amyotrophic Lateral Sclerosis OR Guam Disease OR Amyotrophic Lateral Sclerosis-Parkinsonism-Dementia Complex 1 OR Amyotrophic Lateral Sclerosis Parkinsonism Dementia Complex 1 OR Guam Form of Amyotrophic Lateral Sclerosis) | 86445 |
|  | #3 TS= (Rat OR Mice OR Mouse OR Animal) | 5259652 |
|  | #4 #1 AND #2 AND #3 | 26 |
| 5) Scopus | #1 TITLE-ABS-KEY (Acupuncture OR Electroacupuncture OR "Electric acupuncture" OR Electro-acupuncture OR Acupoint) | 71720 |
|  | #2 TITLE-ABS-KEY ( "Amyotrophic Lateral Sclerosis" OR ALS OR "Lou Gehrig's Disease" OR "Charcot Disease" OR "Amyotrophic Lateral Sclerosis With Dementia" OR "Dementia With Amyotrophic Lateral Sclerosis" OR "Guam Disease" OR "Amyotrophic Lateral Sclerosis-Parkinsonism-Dementia Complex" OR "Guam Form of Amyotrophic Lateral Sclerosis" ) | 202760 |
|  | #3TITLE-ABS-KEY (Rat OR Mice OR Mouse OR Animal) | 9580145 |
|  | #4 #1 AND #2 AND #3 | 36 |
| 6) CNKI | #1 篇关摘：针刺 + 电针 + 针灸 +穴位 | 31549 |
|  | #2 篇关摘：肌萎缩侧索硬化症 + 渐冻症 + ALS | 7379 |
|  | #3 篇关摘：鼠 + 动物 | 1176802 |
|  | #4 #1 AND #2 AND #3 | 18 |

**SYRCLE’s Risk of Bias (ROB) Tool**

The 10 items were assessed individually, with the ten domains classified as low, unclear, or high risk of bias including sequence generation, baseline characteristics, allocation concealment, random housing, blinding of participants/ personnel, random outcome assessment, blinding of the assessor, incomplete outcome data, selective outcome reporting, and other bias. Blinding of participants/personnel was deemed “not applicable” given the impossibility of adequate blinding for acupuncture practitioners.

(1) Generation ofanimal allocation sequence was random; (2) each group was similar or was at adjusted at baseline; (3) the allocation was adequately concealed; (4) animals were housed at random; (5) both animal breeders and researchers were blinded for the intervention of each animal received; (6) animals were selected randomly for outcome evaluation; (7) outcome evaluator was blinded; (8) the incomplete outcome data were absolutely addressed; (9) reports of the research were free of selective outcome reporting; (10) study was evidently free of other potential issues which may cause bias.

**CAMARADES checklist**

The 10 items were counted separately:1) peer-reviewed journal; 2) temperature control; 3) animals were randomly allocated; 4) blind established model; 5) blinded outcome assessment; 6) reporting of animals excluded from analysis; 7) appropriate animal model; 8) calculation of sample size; 9) statement of compliance with animal welfare regulations; 10) possible conflicts of interest. One point was assigned to each of the ten items on the scale.

Table. Risk of bias assessment

| Study | 1 | 2 | 3 | 4 | 5 | 6 | 7 | 8 | 9 | 10 | Score |
| --- | --- | --- | --- | --- | --- | --- | --- | --- | --- | --- | --- |
| Jing HuaJiang(2011) | ? | L | ? | L | NA | ? | ? | L | L | L | 5 |
| Eun Jin Yang(2010) | ? | L | ? | L | NA | ? | ? | L | L | L | 5 |
| Weijia Zhao(2025) | L | L | ? | L | NA | ? | L | L | L | L | 7 |
| Shanshan Liu(2025) | L | L | ? | L | NA | ? | L | L | L | L | 7 |
| Lei Sun(2021) | L | L | ? | L | NA | ? | L | L | L | L | 7 |
| Yuanrong Lu(2024) | L | L | ? | L | NA | ? | L | L | L | L | 7 |
| Shilin Wang(2022) | L | L | ? | L | NA | ? | L | L | L | L | 7 |
| Yuanzheng Sun(2020) | L | L | ? | L | NA | ? | L | L | L | L | 7 |
| Yingzhe Sun(2021) | L | L | ? | L | NA | ? | L | L | L | L | 7 |
| Su Su(2019)a | L | L | ? | L | NA | ? | L | L | L | L | 7 |
| Yuanzheng Sun(2016) | L | L | ? | L | NA | ? | L | L | L | L | 7 |
| Su Su(2019)b | L | L | ? | L | NA | ? | L | L | L | L | 7 |
| Ying Guo(2017) | L | L | ? | L | NA | L | L | L | L | L | 8 |
| Yan Wei(2017) | L | L | ? | L | NA | ？ | L | L | L | L | 7 |
| Junyang Liu(2023) | L | L | ? | L | NA | L | L | L | L | L | 8 |
| Xiawei Liu(2021) | L | L | ? | L | NA | L | L | L | L | L | 8 |
| Shilin Wang(2023) | L | L | ? | L | NA | L | L | L | L | L | 8 |

Abbreviations: L, low-risk bias; H, high risk bias; ?, unclear; NA, not applicable.

**Sensitivity** **Analyses**


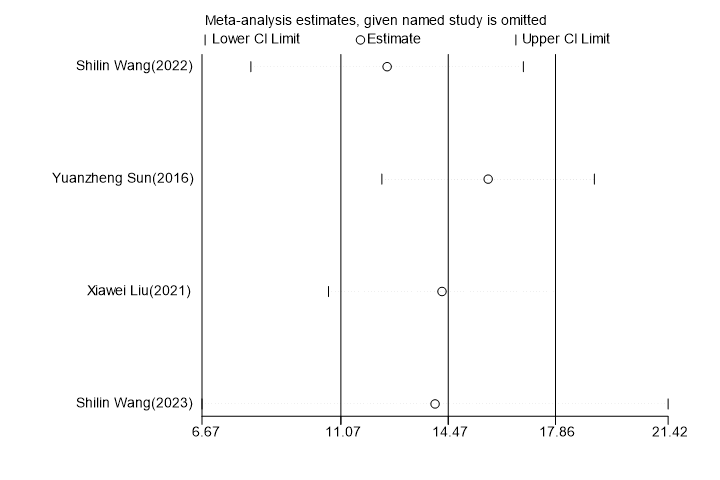


Disease time Sensitivity Analyses


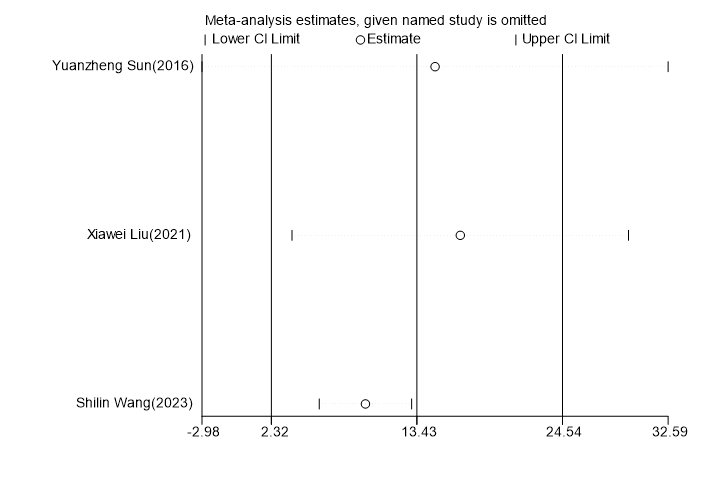


Survival period Sensitivity Analyses


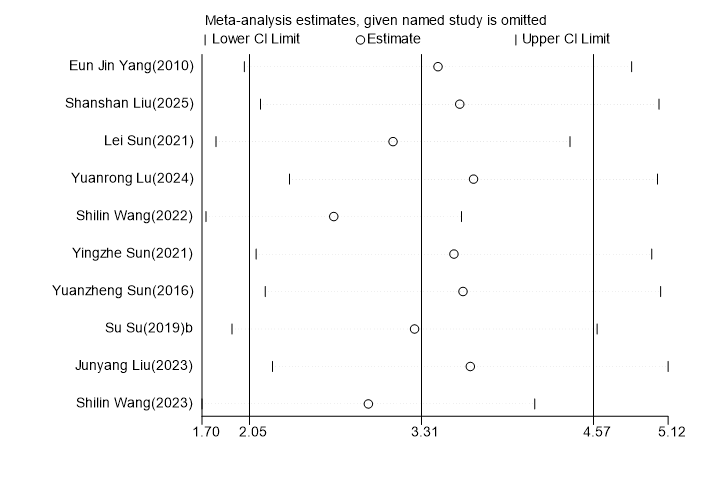


Rotarod test Sensitivity Analyses


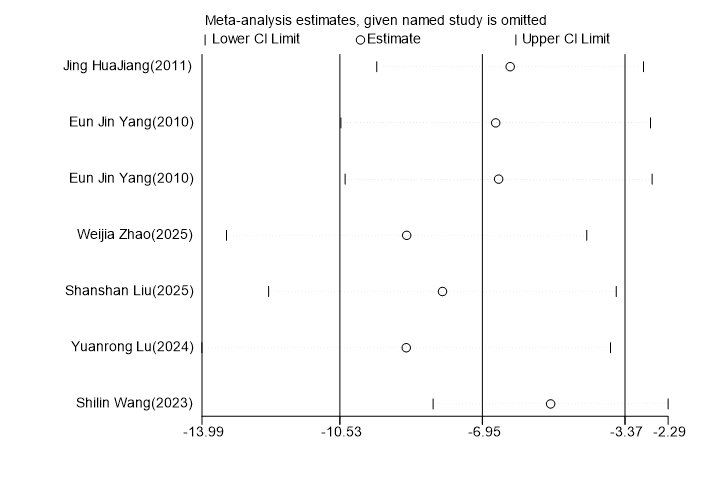


Iba-1 Sensitivity Analyses


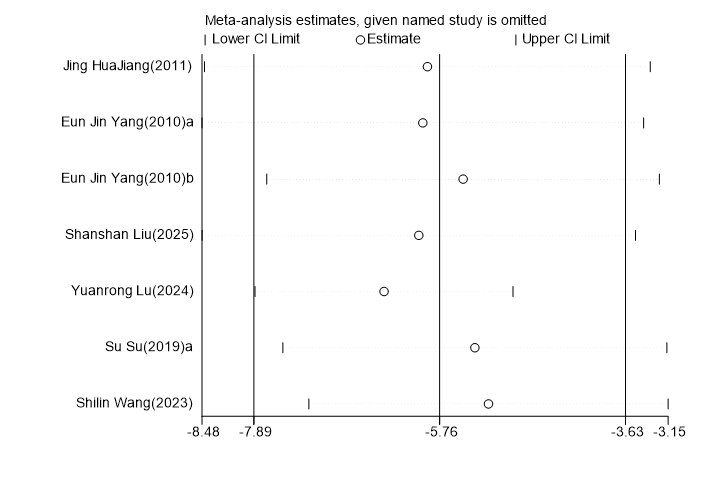


TNF-α Sensitivity Analyses


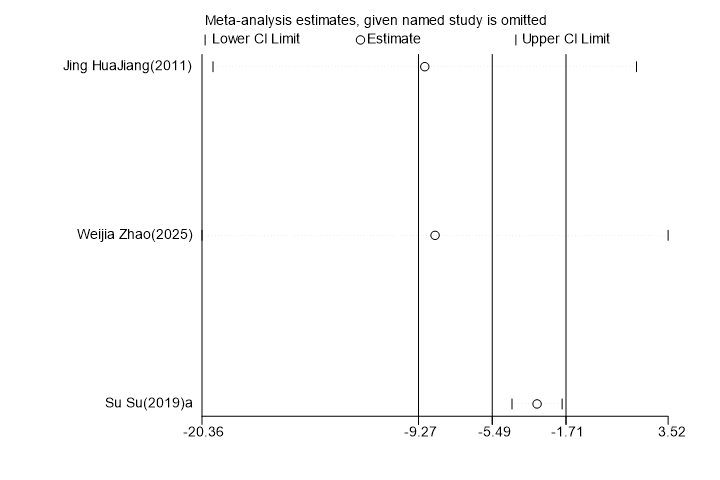


IL-6 Sensitivity Analyses


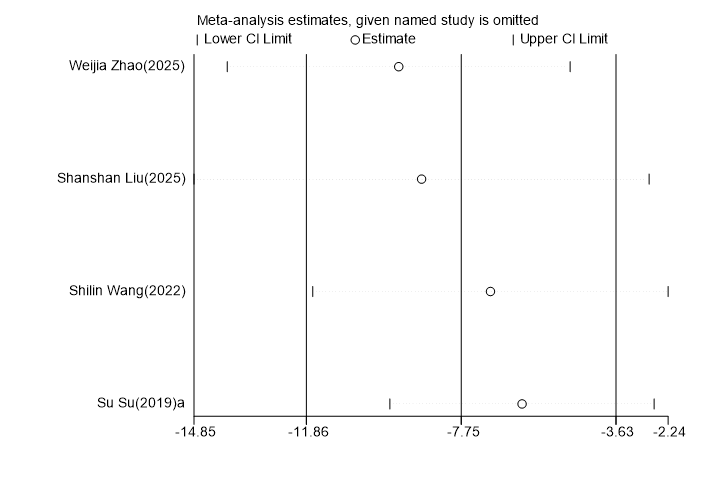


IL-1β Sensitivity Analyses


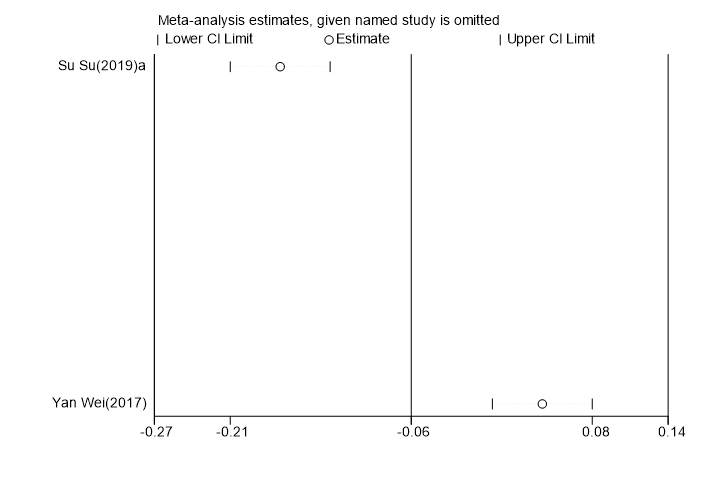


p38 Sensitivity Analyses


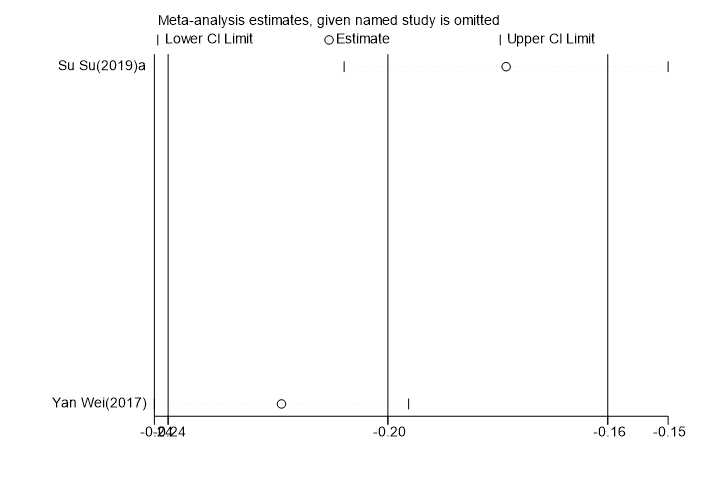


p-p38 Sensitivity Analyses


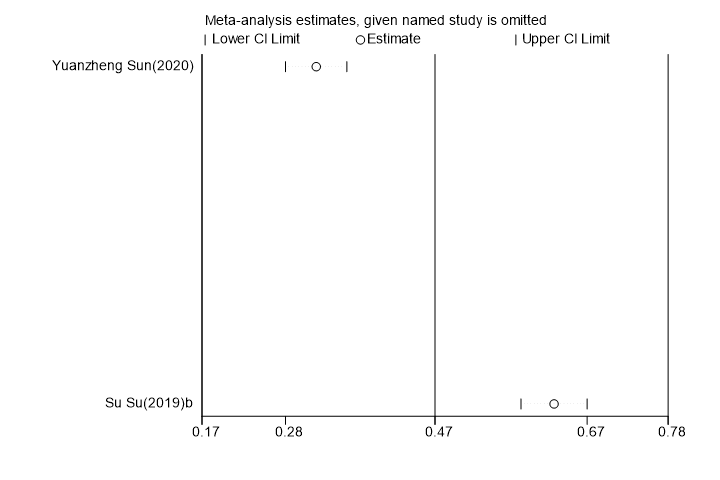


p-GSK-3β Sensitivity Analyses

**Subgroup Analyses**


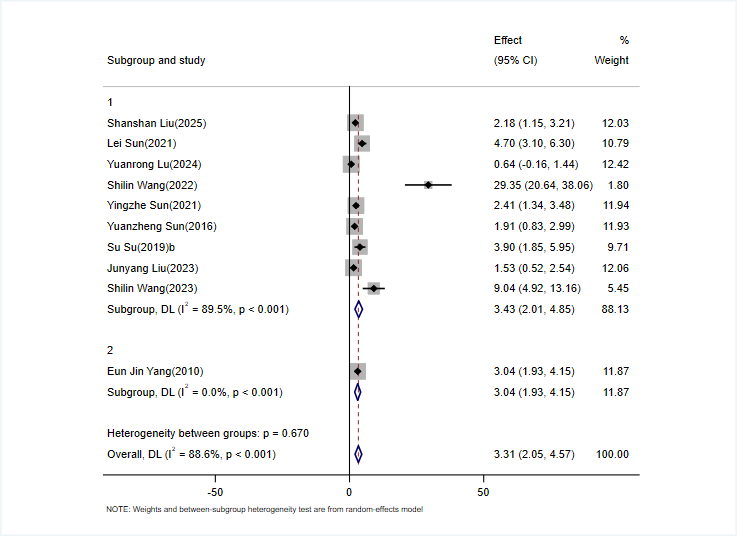


Country-Subgroup Analyses


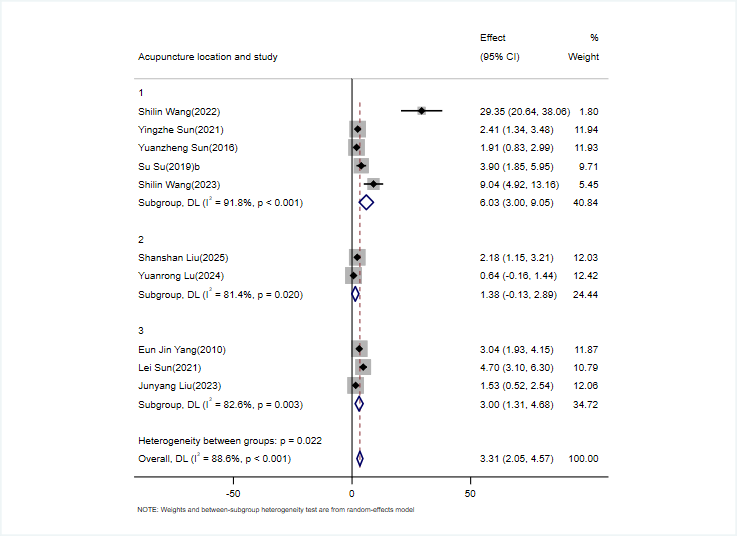


Acupuncture location-Subgroup Analyses


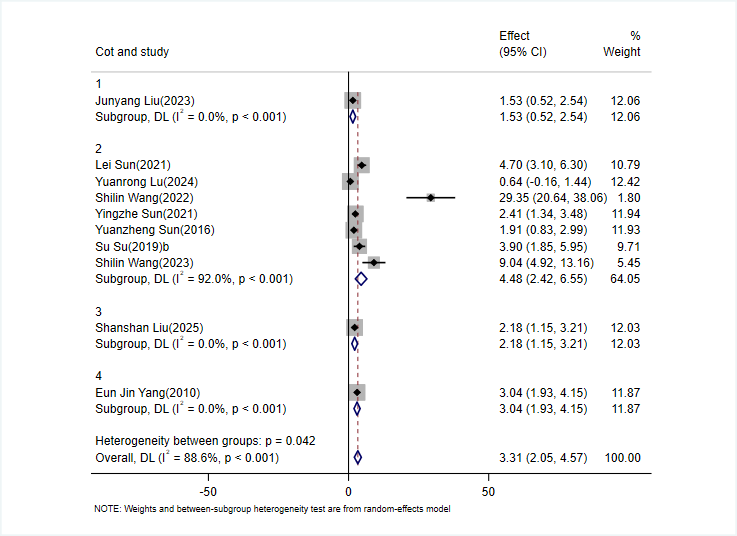


Cot-Subgroup Analyses


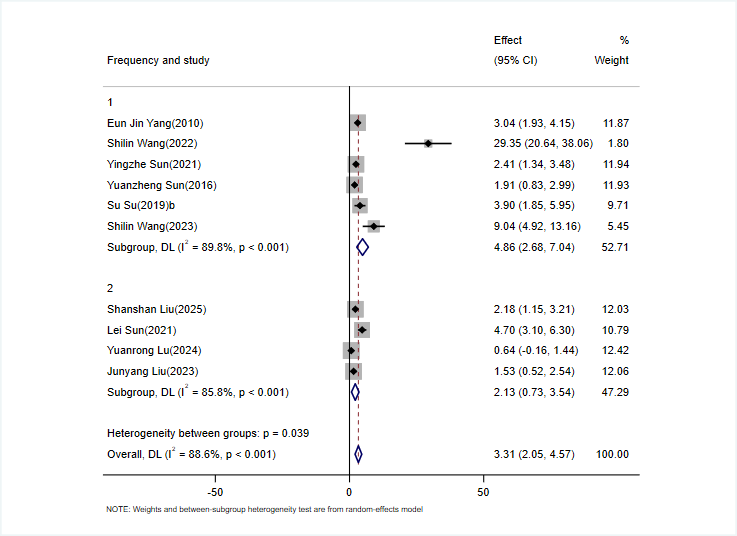


Frequency-Subgroup Analyses


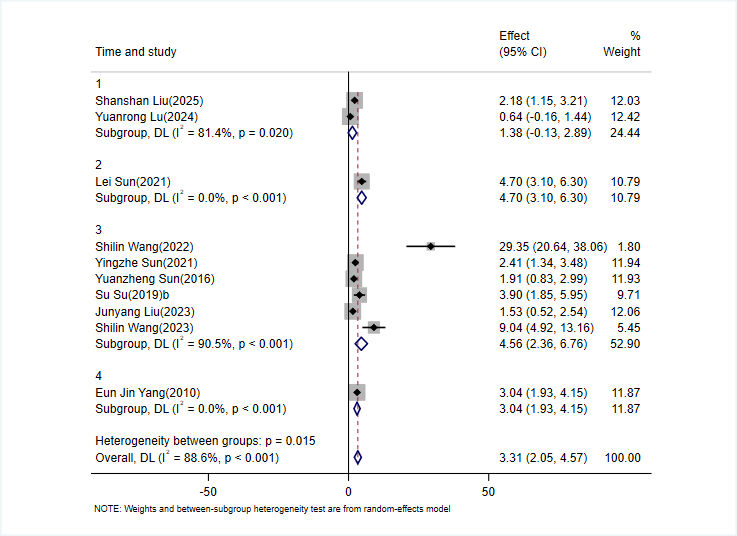


Time-Subgroup Analyses


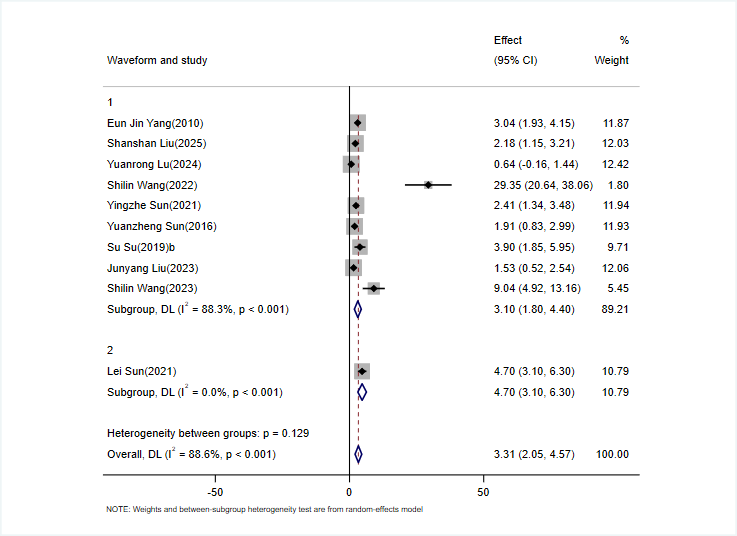


Waveform-Subgroup Analyses


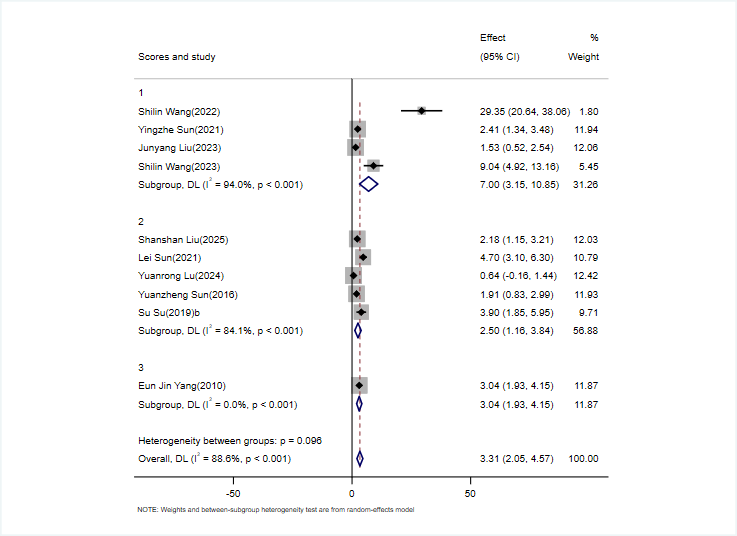


Scores-Subgroup Analyses

**Publication Bias**

**Data Extraction Table**

| bodyweight（g） | EA Group | | | Model Group | | |
| --- | --- | --- | --- | --- | --- | --- |
| study | n1 | mean1 | sd1 | n2 | mean2 | sd2 |
| Lei Sun(2021) | 12 | 21.05 | 0.92 | 12 | 19.47 | 0.38 |
| Yuanzheng Sun(2016) | 10 | 19.7 | 2.17 | 10 | 17.7 | 2.08 |
| Xiawei Liu(2021) | 6 | 18.942 | 1.02 | 6 | 17.99 | 0.98 |
| disease time（d） | EA Group | | | Model Group | | |
| study | n1 | mean1 | sd1 | n2 | mean2 | sd2 |
| Shilin Wang(2022) | 12 | 107.67 | 2.36 | 12 | 90.17 | 0.9 |
| Yuanzheng Sun(2016) | 10 | 102 | 8.165 | 10 | 94.4 | 7.245 |
| Xiawei Liu(2021) | 6 | 107 | 12.1 | 6 | 89.8 | 11.9 |
| Shilin Wang(2023) | 6 | 104 | 1.21 | 6 | 90.17 | 0.9 |
| Survival period(d) | EA Group | | | Model Group | | |
| study | n1 | mean1 | sd1 | n2 | mean2 | sd2 |
| Yuanzheng Sun(2016) | 10 | 152.3 | 4.138 | 10 | 142.2 | 4.417 |
| Xiawei Liu(2021) | 6 | 124 | 4.9 | 6 | 119 | 11.8 |
| Shilin Wang(2023) | 6 | 150.2 | 1.7 | 6 | 127 | 2.5 |
| Rotarod test（s） | EA Group | | | Model Group | | |
| study | n1 | mean1 | sd1 | n2 | mean2 | sd2 |
| Eun Jin Yang(2010) | 15 | 40.18 | 9.13 | 13 | 13.7 | 8.2 |
| Shanshan Liu(2025) | 12 | 77.5 | 23.75 | 12 | 35.63 | 13.12 |
| Lei Sun(2021) | 12 | 180 | 2.71 | 12 | 165 | 3.61 |
| Yuanrong Lu(2024) | 15 | 146.11 | 4.63 | 11 | 143.31 | 3.98 |
| Shilin Wang(2022) | 12 | 168.33 | 4.46 | 12 | 38.17 | 4.41 |
| Yingzhe Sun(2021) | 12 | 57.8 | 26.99 | 12 | 10.62 | 6.27 |
| Yuanzheng Sun(2016) | 10 | 48 | 29 | 10 | 8 | 6 |
| Su Su(2019)b | 6 | 112 | 22 | 6 | 37 | 16 |
| Junyang Liu(2023) | 10 | 57.24 | 15.56 | 10 | 35.93 | 12.12 |
| Shilin Wang(2023) | 6 | 179.12 | 0.88 | 6 | 153.37 | 3.93 |
| Open field test（cm） | EA Group | | | Model Group | | |
| study | n1 | mean1 | sd1 | n2 | mean2 | sd2 |
| Weijia Zhao(2025) | 8 | 2831.62 | 412.37 | 8 | 1841.92 | 357.39 |
| Shanshan Liu(2025) | 12 | 2550 | 650 | 12 | 1575 | 350 |
| Junyang Liu(2023) | 10 | 3556.18 | 421.35 | 10 | 2359.55 | 657.3 |
| Iba-1 | EA Group | | | Model Group | | |
| study | n1 | mean1 | sd1 | n2 | mean2 | sd2 |
| Jing HuaJiang(2011) | 3 | 55.714 | 1.7 | 3 | 104.286 | 0.02 |
| Eun Jin Yang(2010) | 5 | 8.051 | 0.565 | 5 | 20.763 | 2.048 |
| Eun Jin Yang(2010) | 5 | 6.961 | 0.709 | 5 | 29.263 | 3.54 |
| Weijia Zhao(2025) | 5 | 29.495 | 5.274 | 5 | 38.292 | 6.329 |
| Shanshan Liu(2025) | 3 | 7.92 | 0.48 | 3 | 13.92 | 1.92 |
| Yuanrong Lu(2024) | 5 | 1.11 | 0.04 | 5 | 1.27 | 0.05 |
| Shilin Wang(2023) | 6 | 1.74 | 0.18 | 6 | 14.12 | 0.59 |
| TNF-α | EA Group | | | Model Group | | |
| study | n1 | mean1 | sd1 | n2 | mean2 | sd2 |
| Jing HuaJiang(2011) | 5 | 59.09 | 10.6 | 5 | 216.67 | 39.4 |
| Eun Jin Yang(2010)a | 4 | 12.37 | 1.53 | 4 | 24.42 | 2.96 |
| Eun Jin Yang(2010)b | 4 | 2.75 | 0.41 | 4 | 8.24 | 0.88 |
| Shanshan Liu(2025) | 3 | 0.786 | 0.066 | 3 | 1.224 | 0.114 |
| Yuanrong Lu(2024) | 5 | 21.67 | 1.24 | 5 | 24.48 | 1.25 |
| Su Su(2019)a | 6 | 0.30 | 0.06 | 6 | 0.79 | 0.06 |
| Shilin Wang(2023) | 6 | 0.51 | 0.021 | 6 | 0.88 | 0.052 |
| IL-6 | EA Group | | | Model Group | | |
| study | n1 | mean1 | sd1 | n2 | mean2 | sd2 |
| Jing HuaJiang(2011) | 6 | 68.31 | 18.1 | 6 | 126.63 | 23.87 |
| Weijia Zhao(2025) | 6 | 19.04 | 5.03 | 6 | 41.55 | 6.53 |
| Su Su(2019)a | 6 | 0.28 | 0.02 | 6 | 0.85 | 0.05 |
| IL-1β | EA Group | | | Model Group | | |
| study | n1 | mean1 | sd1 | n2 | mean2 | sd2 |
| Weijia Zhao(2025) | 6 | 18.83 | 5.02 | 6 | 41.42 | 6.27 |
| Shanshan Liu(2025) | 3 | 0.82 | 0.03 | 3 | 1.19 | 0.09 |
| Shilin Wang(2022) | 6 | 0.47 | 0.02 | 6 | 0.86 | 0.05 |
| Su Su(2019)a | 6 | 0.36 | 0.02 | 6 | 0.97 | 0.06 |
| NF-κB | EA Group | | | Model Group | | |
| study | n1 | mean1 | sd1 | n2 | mean2 | sd2 |
| Jing HuaJiang(2011) | 6 | 56.94 | 9.19 | 6 | 97.35 | 8.57 |
| Shilin Wang(2022) | 6 | 0.65 | 0.025 | 6 | 0.9 | 0.038 |
| Junyang Liu(2023) | 3 | 0.83 | 0.13 | 3 | 1.53 | 0.15 |
| Shilin Wang(2023) | 6 | 0.64 | 0.03 | 6 | 0.89 | 0.04 |
| TLR4 | EA Group | | | Model Group | | |
| study | n1 | mean1 | sd1 | n2 | mean2 | sd2 |
| Shilin Wang(2022) | 6 | 0.6 | 0.04 | 6 | 1.21 | 0.17 |
| Shilin Wang(2023) | 6 | 0.59 | 0.04 | 6 | 1.2 | 0.17 |
| p38 | EA Group | | | Model Group | | |
| study | n1 | mean1 | sd1 | n2 | mean2 | sd2 |
| Su Su(2019)a | 6 | 0.54 | 0.03 | 6 | 0.5 | 0.04 |
| Yan Wei(2017) | 6 | 0.21 | 0.04 | 6 | 0.38 | 0.03 |
| p-p38 | EA Group | | | Model Group | | |
| study | n1 | mean1 | sd1 | n2 | mean2 | sd2 |
| Su Su(2019)a | 6 | 0.22 | 0.02 | 6 | 0.44 | 0.02 |
| Yan Wei(2017) | 6 | 0.13 | 0.02 | 6 | 0.31 | 0.03 |
| p-GSK-3β | EA Group | | | Model Group | | |
| study | n1 | mean1 | sd1 | n2 | mean2 | sd2 |
| Yuanzheng Sun(2020) | 6 | 0.79 | 0.05 | 6 | 0.16 | 0.02 |
| Su Su(2019)b | 6 | 0.65 | 0.04 | 6 | 0.33 | 0.03 |
| p-Akt | EA Group | | | Model Group | | |
| study | n1 | mean1 | sd1 | n2 | mean2 | sd2 |
| Jing HuaJiang(2011) | 6 | 88.7 | 9.62 | 6 | 34.73 | 9.63 |
| Yuanzheng Sun(2020) | 6 | 0.79 | 0.09 | 6 | 0.41 | 0.08 |
| RhoA | EA Group | | | Model Group | | |
| study | n1 | mean1 | sd1 | n2 | mean2 | sd2 |
| Shanshan Liu(2025) | 3 | 0.72 | 0.08 | 3 | 1.05 | 0.17 |
| Yuanrong Lu(2024) | 5 | 1.02 | 0.2 | 5 | 1.34 | 0.13 |
| TDP-43 | EA Group | | | Model Group | | |
| study | n1 | mean1 | sd1 | n2 | mean2 | sd2 |
| Shanshan Liu(2025) | 3 | 0.63 | 0.08 | 3 | 1.04 | 0.15 |
| Yuanrong Lu(2024) | 5 | 0.55 | 0.05 | 5 | 0.75 | 0.11 |
| Junyang Liu(2023) | 3 | 0.91 | 0.08 | 3 | 1.11 | 0.07 |
| amount of motor neurons | EA Group | | | Model Group | | |
| study | n1 | mean1 | sd1 | n2 | mean2 | sd2 |
| Shilin Wang(2022) | 6 | 16.5 | 1.12 | 6 | 9.33 | 1.49 |
| Ying Guo(2017) | 6 | 14.83 | 2.99 | 6 | 8.5 | 1.64 |
